# Improving rice eating and cooking quality by enhancing endogenous expression of a nitrogen‐dependent floral regulator

**DOI:** 10.1111/pbi.14160

**Published:** 2023-08-25

**Authors:** Yuyi Zhang, Shunan Zhang, Jinfei Zhang, Wei Wei, Tao Zhu, Hongye Qu, Ying Liu, Guohua Xu

**Affiliations:** ^1^ National Key Laboratory of Crop Genetics & Germplasm Enhancement and Utilization Nanjing Agricultural University Nanjing China; ^2^ Key Laboratory of Plant Nutrition and Fertilization in Low‐Middle Reaches of the Yangtze River, Ministry of Agriculture Nanjing Agricultural University Nanjing China; ^3^ State Key Laboratory of Pharmaceutical Biotechnology, School of Life Sciences Nanjing University Nanjing China

**Keywords:** amino acids metabolism, carbohydrate metabolism, circadian clock, eating and cooking quality (ECQ), nitrogen‐regulated heading date‐1 (Nhd1), N use efficiency (NUE)

## Abstract

Improving rice eating and cooking quality (ECQ) is one of the primary tasks in rice production to meet the rising demands of consumers. However, improving grain ECQ without compromising yield faces a great challenge under varied nitrogen (N) supplies. Here, we report the approach to upgrade rice ECQ by native promoter‐controlled high expression of a key N‐dependent floral and circadian clock regulator Nhd1. The amplification of endogenous Nhd1 abundance alters rice heading date but does not affect the entire length of growth duration, N use efficiency and grain yield under both low and sufficient N conditions. Enhanced expression of Nhd1 reduces amylose content, pasting temperature and protein content while increasing gel consistence in grains. Metabolome and transcriptome analyses revealed that increased expression of Nhd1 mainly regulates the metabolism of carbohydrates and amino acids in the grain filling stage. Moreover, expression level of *Nhd1* shows a positive relationship with grain ECQ in some local main cultivars. Thus, intensifying endogenous abundance of Nhd1 is a promising strategy to upgrade grain ECQ in rice production.

## Introduction

Rice is the staple food for more than half of the world's population. Ensuring sufficient grain yield to feed the growing population is a primary target in rice production. With the increase of people's living standards, improving rice quality is also highly demanded. Rice grain quality is defined by multiple traits, including appearance, nutrients, cooking and eating property (Liu *et al*., 2022; Sattari *et al*., 2015; Zahra *et al*., 2022). The preference for eating and cooking quality (ECQ) of rice exhibits regional differences, namely people from south Asia prefer aromatic rice which is fluffy and dry after cooking, while people from northeast Asia, such as China, Korea and Japan, prefer *japonica* rice cultivars with softness and stickiness taste (Costa de Oliveira *et al*., 2020; Custodio *et al*., 2019; Hori *et al*., 2021).

Eating and cooking quality of rice is mainly determined by two major components in grains, starch and protein, which occupy approximately 80%~85% and 4%~10% proportion of grain biomass, respectively (Balindong *et al*., 2018). Starch in rice grain is composed of amylose and amylopectin, serving as the dominant factor of cooking property and eating texture (Bao, 2012; Pandey *et al*., 2012). Specifically, rice with higher amylose content (AC) (>25%) is fluffy on cooking and gets hard after cooling, while rice with lower AC becomes softer and stickier after cooking (Custodio *et al*., 2016, 2019; Sattari *et al*., 2015). In this context, amylopectin with more branching structure endows rice with softness taste (Ramesh *et al*., 1999). Four types of enzymes, including ADP‐glucose phosphorylase (AG‐Pase), starch synthase (SS), starch branching enzyme (SBE) and starch debranching enzyme (DBE), coordinate together to facilitate starch synthesis. These genes play a distinct role in starch synthesis, such as *Wx* for amylose synthesis, *SSI*‐*SSIV* for amylopectin chain‐elongation, *SBEs* for new branch introduction and *ISA* for improper branch removement (Aoki *et al.,* 2006; Chen *et al*., 2012; Tian *et al*., 2009; Wang *et al*., 1995). Grain storage protein (GSP) is synthesized by amino acids imported from source tissues. 60% to 80% of GSP is in the form of glutelin, which is synthesized by 15 genes belonging to four groups (GluA, GluB, GluC and GluD) (Kawakatsu *et al*., 2008, 2010; Tegeder and Ward, 2012). The protein content in grain has been reported to be negatively related to cooking property, and more amino acids also lead to the deterioration of rice eating taste (Baxter, 2004, 2013; Nakamura *et al*., 2016). Notably, ECQ is an integrated trait influenced by the structure and content of starch and protein. Despite of similar AC, different proportion of amylopectin long chains and protein content can lead to an altered ECQ (Han and Hamaker, 2001; He *et al*., 2010; Peng *et al*., 2021). Therefore, rapid visco analyser (RVA), which generates three 1st‐grade indicators (peak viscosity, hot paste viscosity and cool paste viscosity), three 2nd‐grade indicators (breakdown and setback) and a parameter of pasting temperature, is widely used to dissect a comprehensive profile of rice ECQ (Champagne *et al*., 1999; Zhang *et al*., 2019).

Nitrogen (N) fertilization largely impacts rice yield and quality. In general, more N fertilization increases yield but deteriorates ECQ (Cao *et al*., 2018). Increasing N application leads to high‐protein content and even a change of protein secondary structure in grains, thus negatively affecting rice cooking and gelatinization properties (Liang *et al*., 2021). N supply also affects starch properties by regulating activities of starch synthesis enzymes (Wei *et al*., 2018; Zhou *et al*., 2020). Notably, elevating N input can decrease apparent amylose content (AAC), whereas some rice cultivars show insensitivity of AAC to N supply level, suggesting that the response of starch composition as well as rice ECQ to N application shows genetic diversity (Huang *et al*., 2020b; Tang *et al*., 2019).

A complete rice growth season is divided to vegetative, reproductive and grain‐filling stages (Fageria *et al*., 2006). Appropriate elongation of growth duration can commonly increase grain yield, while the effect of growth period on grain quality is more complex and shows the interaction between multiple genetic and environmental factors (Krishnan *et al*., 2011; Lang *et al*., 2012; Lin *et al*., 2020). 70%–90% of grain N is transferred from the product accumulated before leaf senescence and remobilized from source tissues during reproductive stage, while the rest N in grains (10%–30%) is absorbed from the soil or late foliar fertilizers (Mae, 1997; Yoshida, 1972). At the reproductive stage, many amino acid transporters are induced to facilitate N remobilization from source tissue to grains, in turn, the storage proteins in grain are predominantly synthesized in maturing endosperm tissue (Kawakatsu and Takaiwa, 2010; Masclaux‐Daubresse *et al*., 2008). Differently, approximately 75% of carbon (C) in grain is generated from photosynthesis during grain filling stage (Yoshida, 1981). The length of grain filling stage is tightly associated with the accumulation of both starch and protein, thus, it is critical for grain quality formation (Zhang *et al*., 2021c). Although shortening grain filling stage usually leads to poor ECQ, an extension of this stage not always improves ECQ. Excessive prolongation of filling stage increases the risk of rice being exposed to an inappropriate environment condition, for example, the abnormal temperature, thus impairing both yield and ECQ (Krishnan *et al*., 2011).

We have previously identified a floral regulator belonging to MYB transcription factor family in rice, N‐mediated‐heading‐date‐1 (Nhd1), which regulates flowering time and coordinates carbohydrate and N metabolisms (Li *et al*., 2022a,b; Zhang *et al*., 2021d). Nhd1, also named OsCCA1 (Wang *et al*., 2020), acts as a circadian clock regulator and directly activates the expression of floral gene *OsHd3a* to promote flowering in rice (Zhang *et al*., 2021d). In addition, Nhd1 modulates N uptake and distribution via activating ammonium transporter *OsAMT1;3* and nitrate transporter *OsNRT2.4*, as well as N assimilation by inhibiting Ferredoxin‐dependent glutamate synthase (*Fd‐GOGAT*) and sucrose distribution by targeting sucrose transporter *OsSUT1* (Li *et al*., 2022a; Zhang *et al*., 2021d). These evidences suggest that Nhd1 as a key integrator to coordinate growth duration and C and N utilization may also play a critical role in the regulation of grain ECQ in rice.

In this study, we have demonstrated that enhanced expression of Nhd1 driven by its native promoter can improve rice ECQ under both low and high‐N supplies. The Nhd1 over‐expression reduces the N‐controlled alteration of heading date but it neither affects grain yield nor N use efficiency. Nhd1 regulates ECQ‐related processes at transcriptional level, leading to the coordination of carbohydrate and amino acid metabolisms for better ECQ in grain filling stage.

## Results

### Enhancing expression of Nhd1 by its native promoter weakens N‐dependent change of heading date in rice

As a key circadian and floral regulator in rice, the function of Nhd1 is largely determined by its uniquely spatio‐temporal expression pattern (Wang *et al*., 2020; Zhang *et al*., 2021d). To intensify the abundance of *Nhd1* without altering its original expression pattern and rhythm, a native promoter strategy for gene over‐expression was chosen in this study. In this context, an Nhd1‐coding construction driven by a 2.5 kb native promoter sequence upstream of *Nhd1* transcriptional start site (*pNhd1::Nhd1*) was introduced into wild‐type (WT) rice to generate *Nhd1* over‐expression (OX) plants, and 11 transgenic lines were successfully obtained (Figure S1a). Based on Southern blot analysis, three independent Nhd1‐OX lines (OX1, OX2 and OX3) were selected for detailed analysis (Figure S1b). Compared with WT, these transgenic lines show an increase of *Nhd1* transcript abundance by twofold to eightfold and its protein level by twofold to fivefold in both root and shoot (Figure S1c,d). Notably, circadian rhythm expression pattern of *Nhd1* was unchanged in the Nhd1‐OX lines (Figure S1e).

**Figure 1:**
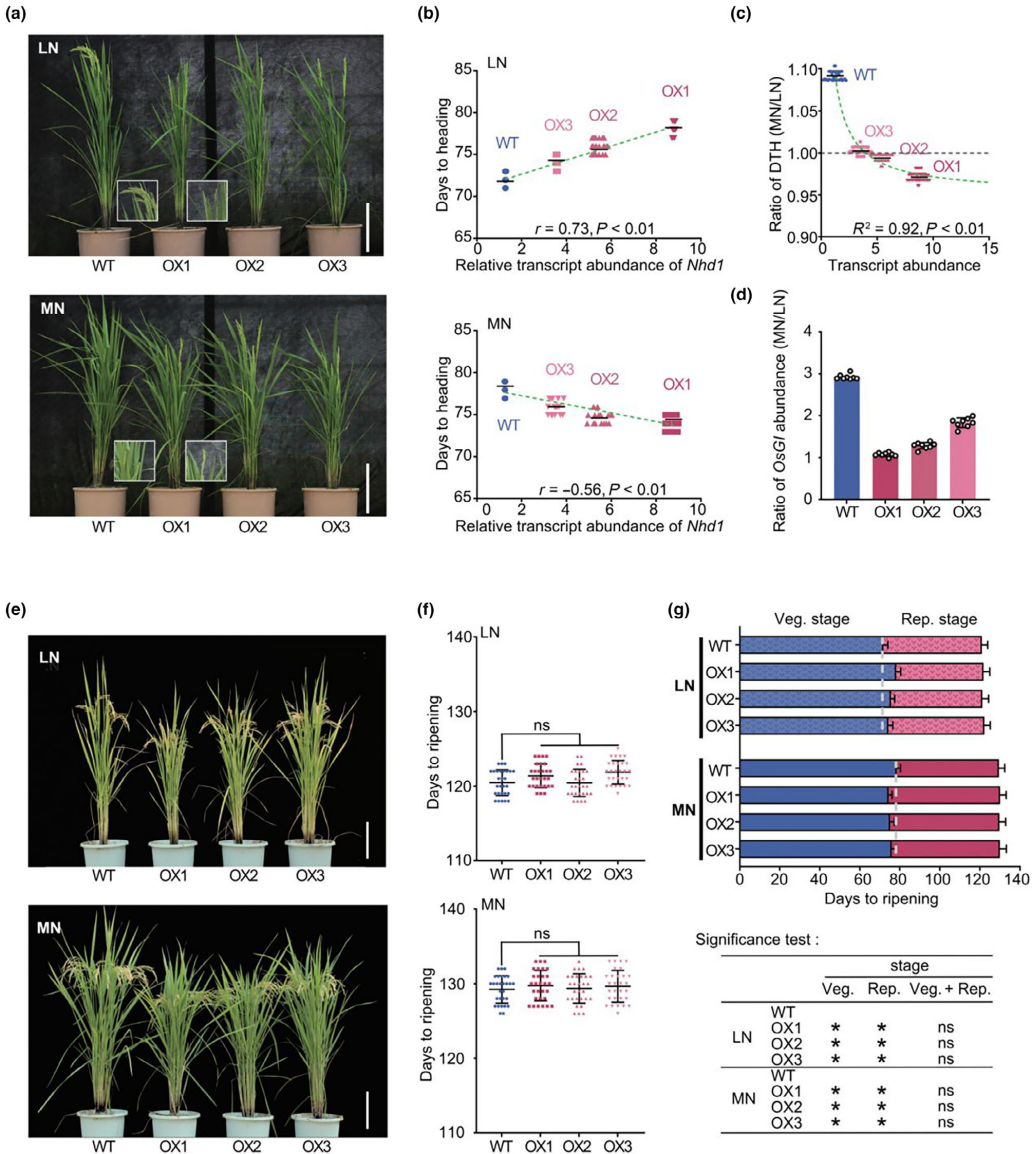
Over‐expression of *Nhd1* reduces the sensitivity of heading date to different N supplies and do not change the length of entire growth duration. (a) Phenotype of wild‐type (WT) and *pNhd1:Nhd1* transgenic plants (Nhd1‐OX1, OX2 and OX3) under different N treatments at heading stage. N was supplied at 150 kg/ha for low N (LN) or 250 kg/ha for moderate N (MN). (b) Days to heading (DTH) after transplanting of WT and Nhd1‐OX lines under LN and MN treatments. And the correlations between *Nhd1* transcript abundance and days to heading (DTH) in WT and Nhd1‐OX lines under LN and MN treatments. DTH is defined as the days from transplanting to heading. (c) The correlations between *Nhd1* transcript abundance and ration of DTH in MN to that in LN. (d) The ratio of *OsGI* transcript abundance in MN to that in LN treatment in WT and Nhd1‐OX plants. (e) Phenotype of WT and Nhd1‐OX plants at ripening stage in LN and MN conditions. (f) Days to ripening of WT and Nhd1‐OX plants under LN and MN treatments. It is defined as the days from transplanting to ripening. (g) Comparison of vegetative (Veg. stage) and reproductive & ripening stage (Rep. Stage) between WT and Nhd1‐OX plants. Multiple comparisons were used for significance test between WT and each Nhd1‐OX line. Asterisk shows a significant difference at *P* < 0.01. Two‐tailed Student's *t*‐test was used for the statistical analysis in (b), (c), (d), (f) and (g). The values represent means ± SD. Spots with different colours represent the value of each biological replicates (*n* > 30). The green line represents the correlation fitting curve, and the *R* ^2^, *r*‐ and *P*‐value of each curve has been given.

A critical function of Nhd1 in rice is its regulation of N‐mediated heading (flowering) (Zhang *et al*., 2021d). To elucidate the impact of enhanced Nhd1 expression on heading responding to N supply, field trials of three Nhd1‐OX lines under low N (150 kg/ha; LN) or moderate N (250 kg/ha; MN) have been implemented for 2 years. Interestingly, over‐expression of Nhd1 delayed heading under LN supply but promoted it under MN supply (Figures 1a, S2a). Consistently, the heading date was closely correlated with the transcript and protein abundance of Nhd1 in all treatments (Figures 1b, S2c), revealing that the changes in heading date in Nhd1‐OX lines are caused by the altered expression of *Nhd1*. This phenomenon was confirmed by field trial in the second growth season with a similar performance (Figure S3a).

To better evaluate the response of heading time to the changing N supplies, a heading date ratio between MN and LN treatment was calculated. As shown, the heading date ratio (MN / LN) in WT was in proximity to 1.1, suggesting a slightly delayed heading time in WT upon elevated N input; whereas this ratio in Nhd1‐OX lines was close to 1.0 (Figure S2b), implying a decreased sensitivity of heading time in response to change of N inputs in Nhd1‐OX lines compared with that in WT. In addition, the transcription and protein levels of Nhd1 were tightly correlated to the heading date ratio (Figures 1c, S2d), confirming that enhancing Nhd1 expression reduced the N‐regulated change of heading time.

To confirm Nhd1‐overexpression caused converted alteration of heading time by N applications, we detected expression of *OsGI*, a key integrator between the circadian clock and flowering that also serves as a typical molecular indicator of heading process (Brambilla and Fornara, 2013; Shin *et al*., 2004; Sun *et al*., 2021). As expected, *OsGI* expression in Nhd1‐OX lines was upregulated under LN but repressed under MN (Figure S2e). Consistently, the change of *GI* expression responding to N supply was less sensitive in Nhd1‐OX lines than that in WT (Figure 1d), as similar as the response of heading date to LN and MN conditions. Hence, these results reveal that the highly expression of Nhd1 can stabilize rice heading date under varied N applications.

### Enhancing expression of Nhd1 does not affect growth duration and grain yield

Heading date defines the transition process from vegetative to reproductive stage (Takai *et al*., 2006; Zhang *et al*., 2022). When we made a comparison between WT and Nhd1‐OX lines across the entire growth season, it was interesting to see that Nhd1‐OX lines underwent a longer vegetative growth but a shorter reproductive stage than WT plants under LN supply, while this pattern was reversed under MN condition, finally leading to an unchanged growth duration between Nhd1‐OX lines and WT under both LN and MN conditions (Figures 1e–g, S3b).

**Figure 2:**
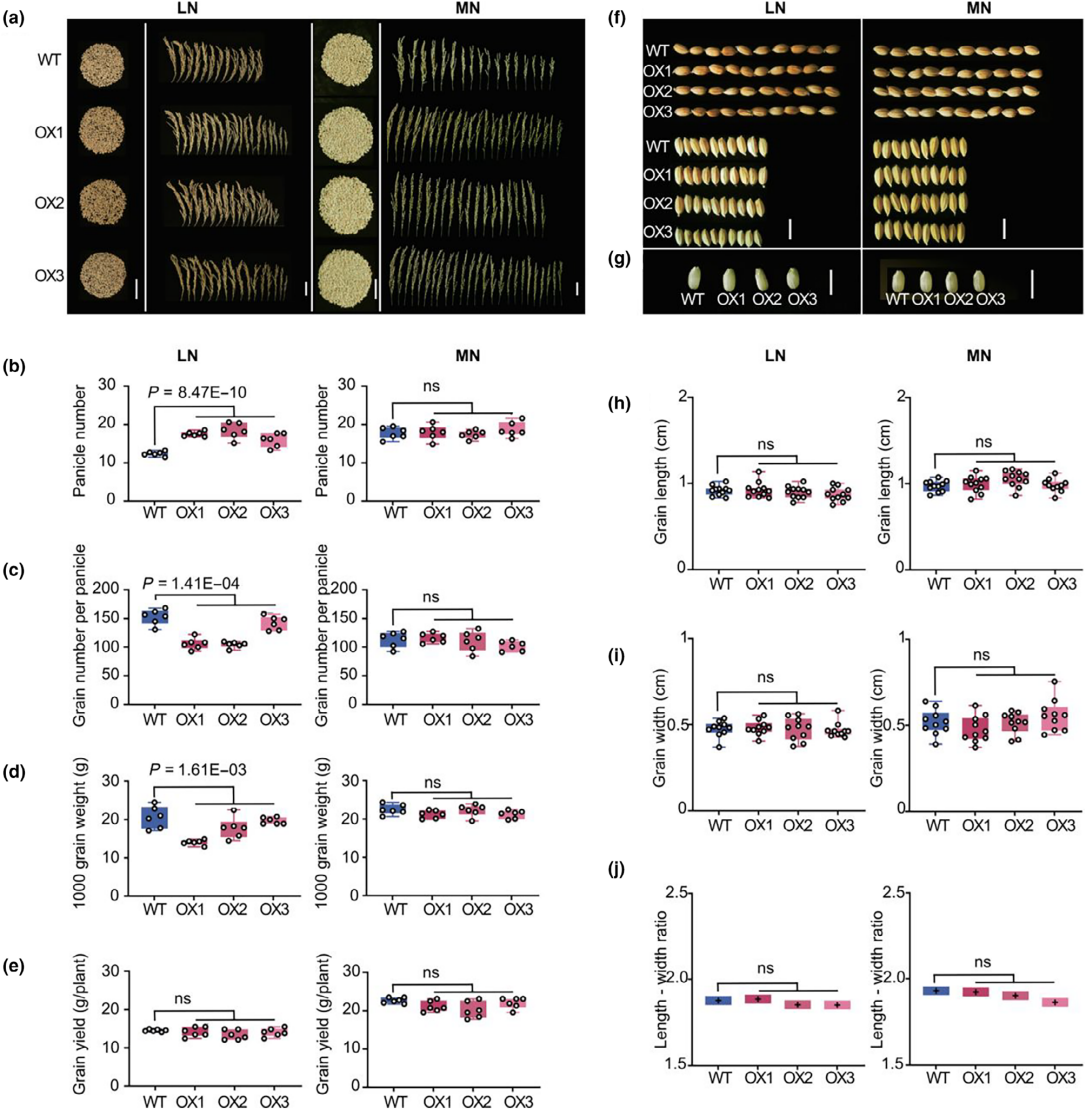
Grain yield and appearance quality of WT and Nhd1‐OX lines in different N conditions. (a) Phenotype of yield for a single plant and the fruiting condition for each panicle. (b–e) Panicle number (b), Grain number per panicle (c), 1000 grain weight (d) and Grain yield (e) of WT and Nhd1‐OX plants in LN and MN conditions. (f, g) Grain appearance of WT and Nhd1‐OX plants under different N treatments. (h–j) comparison of grain length(h), grain width (i), length‐to‐width ratio (j) of WT and Nhd1‐OX plants in LN and MN conditions. Bars are means ± SD, and each point shows a mean value of each plant (*n* ≥ 6). One‐tailed Student's *t*‐test was used for the statistical analysis. *P*‐values are provided in the figures. Ns means no significant difference.

For rice production, grain yield is determined by three major traits, namely panicle number, grain number per panicle and thousand grain weight (Takai *et al*., 2006; Xing and Zhang, 2010). In our field trials, we found that enhancing expression of *Nhd1* led to an increase of panicle number but a decrease of grain number per panicle, thousand grain weight and seed setting under LN input, finally keeping the same yield with WT at our experimental condition (Figures 2a–e, S4a–c). By contrast, all yield component traits showed no difference between Nhd1‐OX lines and WT under MN supply (Figures 2a–e, S4a–c). Furthermore, a detailed dissection of grain size including grain length, width and the ratio of length to width showed no significant change between Nhd1‐OX lines and WT irrespective of N supply levels (Figure 2f–j). Correspondingly, transcription levels of *SPL16* and *GW7*, two crucial genes related to grain size (Li *et al*., 2018; Wang *et al*., 2015), did not show significant changes in Nhd1‐OX lines compared to that in WT (Figure S4d). Therefore, we conclude that over‐expression of *Nhd1* driven by its native promoter neither alters the entire growth duration nor grain yield in rice.

### Enhancing expression of Nhd1 increases N uptake efficiency while decreasing N utilization efficiency under limited N supply

**Figure 3:**
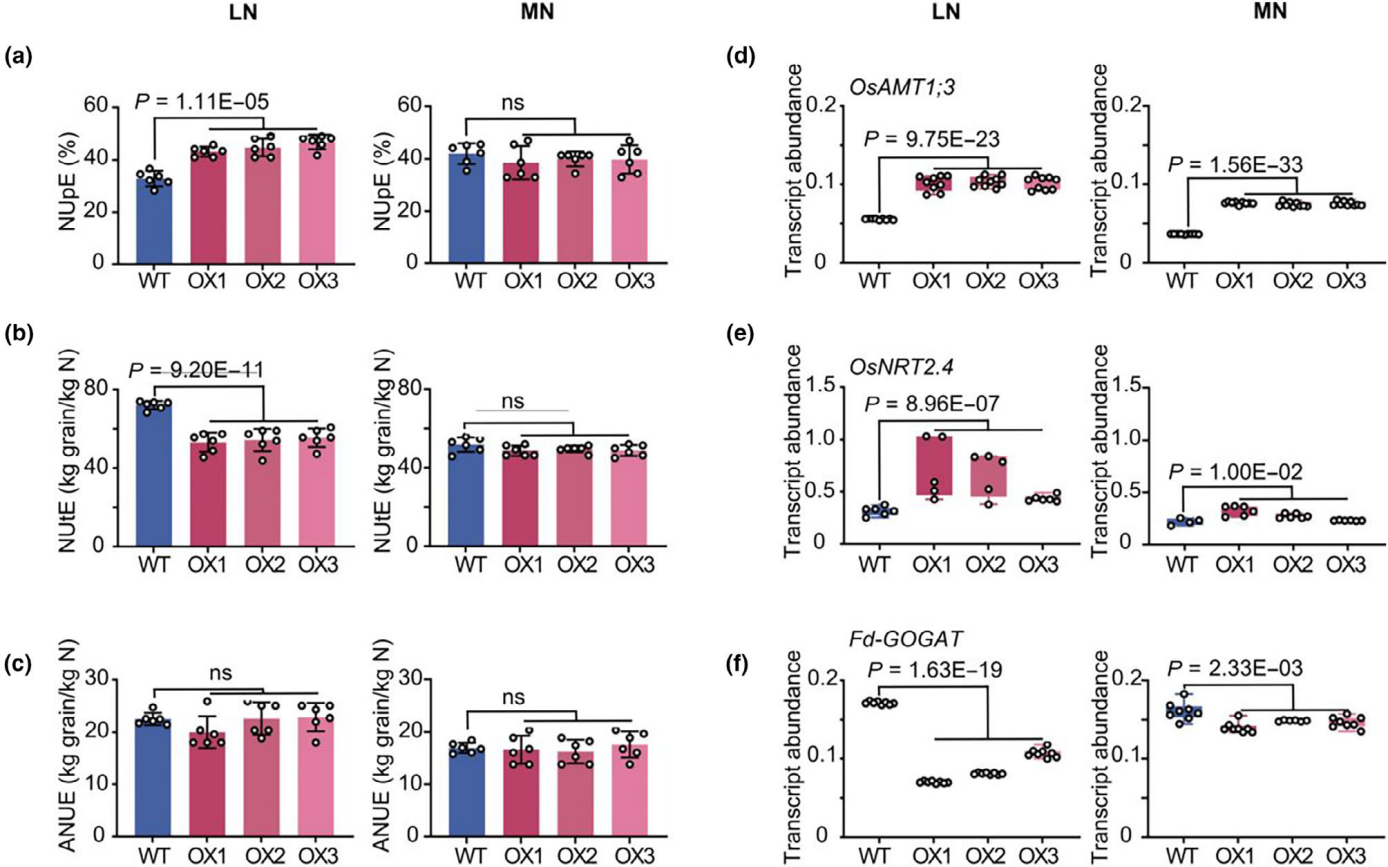
The impact of *Nhd1* over‐expression on N uptake, utilization and N use efficiency under different N supplies. (a–c) N uptake use efficiency (NUpE, a), N utilization efficiency (NUtE, b) and N use efficiency (NUE, c) in WT and Nhd1‐OX plants under different N treatments. (d–f) Relative abundance of Nhd1‐regulated genes related to N uptake and assimilation in WT and Nhd1‐OX plants under different N treatments. Bars are means ± SD, and each point shows a specific value of each plant (*n* ≥ 6 plants). One‐tailed Student's *t*‐test was used for the statistical analysis. The *P*‐values are provided.

N use efficiency (NUE) is mainly characterized by two aspects, namely N uptake efficiency (NUpE) and N utilization efficiency (NUtE). NUpE refers to the percentage of N fertilizer acquired by plant, while NUtE means the fraction of plant‐acquired N to form grain yield (Xu *et al*., 2012). Our previous studies report that Nhd1 can regulate both N uptake and assimilation in rice (Li *et al*., 2022b; Zhang *et al*., 2021d). To address the question of whether enhancing expression of *Nhd1* influenced NUE, we calculated NUpE, NUtE and NUE in all tested lines, showing that NUpE was improved while NUtE was reduced in Nhd1‐OX lines for LN treatment (Figure 3a–c). Interestingly, the loss of NUtE in Nhd1‐OX lines was likely made up by improved NUpE at the same time, thus no significant change of NUE was detected between Nhd1‐OX lines and WT under LN supply (Figure 3a–c). By contrast, these three NUE parameters were not affected by enhancing expression of *Nhd1* under MN supply (Figure 3a–c).

Next, we clarified how over‐expression of *Nhd1* caused the opposite impact on NUpE and NUtE when N supply was limited. NUpE is mainly determined by N uptake activity and root architecture (Kiba and Krapp, 2016; Xu *et al*., 2012). We have previously shown that Nhd1 can directly activate Os*AMT1.3* and Os*NRT2.4* to increase ammonium uptake and lateral root growth under limited N condition (Li *et al*., 2022b). Over‐expression of *Nhd1* especially under LN supply significantly enhanced expression of these two genes in agreement with the improved NUpE at this condition (Figure 3d,e). N assimilation enzyme *OsFd‐GOGAT* is also a direct target of Nhd1 but its expression is negatively regulated by Nhd1 (Yang *et al*., 2016; Zhang *et al*., 2021d). Indeed, *OsFd‐GOGAT* expression was down‐regulated in Nhd1‐OX plants under LN condition (Figure 3f), coinciding with a reduced NUtE. Meanwhile, all the expressions of *OsAMT1.3*, Os*NRT2.4 and OsFd‐GOGAT* were less affected by increasing abundance of *Nhd1* under MN supply, indicating a minor role of Nhd1 in affecting NUE at sufficient N level (Figure 3d–f).

### Enhancing expression of Nhd1 improves ECQ irrespective of N supplies

**Figure 4:**
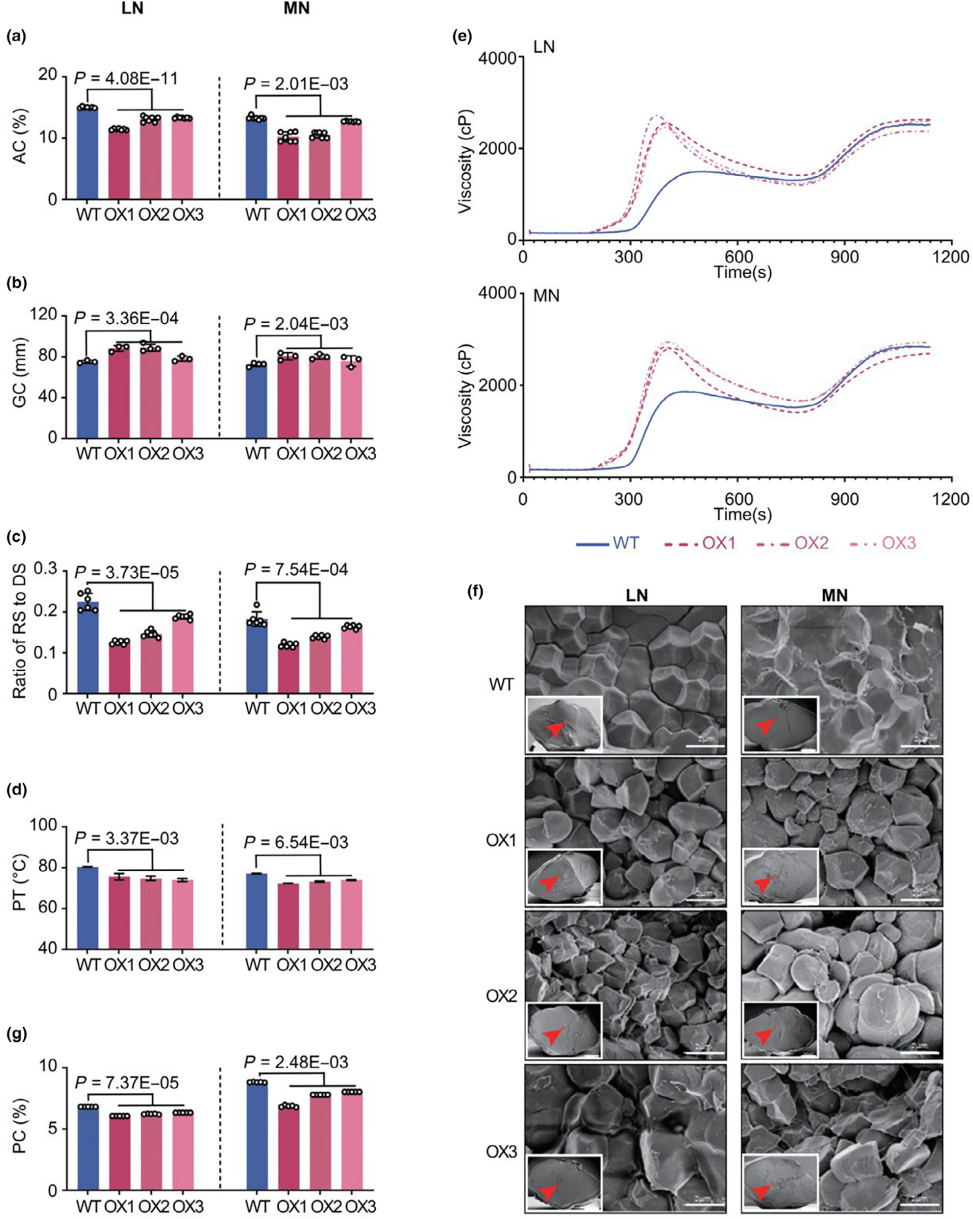
Over‐expression improves grain ECQ in both LN and MN conditions. (a–d, g) Comparison of amylose content (AC) (a), Gel consistency (GC) (b), ratio of resistant starch to digestible starch content (ratio of RS to DS) (c), Pasting temperature (PT) (d), Total protein content (PC) (g) of WT and Nhd1‐OX rice under different N treatments. (e, f) Rapid viscosity analysis (RVA) (e) and starch granule morphology of grain cross section (f) of WT and Nhd1‐OX rice under different N treatments. Bars are means ± SD (a–d, g), and each point shows a specific value of each plant (*n* ≥ 3). One‐tailed Student's *t*‐test was used for the statistical analysis (a–d, g). The *P*‐values are provided, while ns means no significant difference (a–d, g). The red box (f) represents the viewing area of the SEM. Scale bar of the thumbnail is 500 μm, while scale bar of the enlarged image is 2 μm in (f).

C and N are the dominant components in grain that are highly related to grain quality (Balindong *et al*., 2018). Nhd1 has been verified as a key regulator to impact the balance of C and N (Li *et al*., 2022b). In this study, we found that over‐expression of *Nhd1* did not affect N concentration but increased C concentration and a higher ratio of C/N in grains (Figure S5), which led us to make hypothesis that the enhancement of *Nhd1* may affect grain quality, especially ECQ in rice. Thus, we measured the main ECQ properties of both WT and Nhd1‐OX rice grown in different N conditions. Remarkably, over‐expression of *Nhd1* decreased amylose content (AC) by over 15% (Figure 4a) and enhanced soft gel consistence (GC) by proximately 10% in both LN and MN conditions (Figure 4b). As amylose content in raw rice starch is negatively correlated with starch digestibility (Asp and Björck, 1992; Huang *et al*., 2023), we further evaluated the impact of *Nhd1* over‐expression on starch digestion property by measuring the status of resistant starch in grains. Compared with that in WT, the ratio of resistant starch and digested starch decreased in the grains of Nhd1‐OX lines under both LN and MN conditions (Figure 4c), indicating that over‐expression of *Nhd1* improves starch digestibility in grains. Concerning pasting characteristics, the RVA profiling showed rice flours of Nhd1‐OX lines in comparison with WT exhibited a dramatically higher breakdown value (BDV) and a lower setback value (SBV), following an increase of peak viscosity (Figure 4e and Table 1). Moreover, both the pasting time and pasting temperature of grain in Nhd1‐OX plants were also reduced (Figure 4d and Table 1). These results showed the viscosity properties of starch in grain are significantly improved by multiplying the expression of Nhd1. To deeply explore the reasons for better starch properties in Nhd1‐OX grains, we observed the inner polyhedral starch granules of rice kernels by scanning electron microscope (SEM). Even though both the WT and Nhd1‐OX lines had typical angular‐shaped compound‐type starch granules, starch granules in WT were compactly arranged, whereas starch granules of Nhd1‐OX lines were arranged more loosely, irrespective of N supply conditions (Figure 4f). Such a loose structure of starch granules endows the grains of Nhd1‐OX lines with better cooking properties and digestibility (Fu and Xue, 2010; Zhang *et al*., 2019).

**Table 1:** Pasting characteristics and thermal properties of WT and Nhd1‐OX rice under LN and MN conditions

	Pat (s)	PaT (°C)	PKT (s)	PKV (cP)	HPV (cP)	CPV (cP)	BDV (cP)	SBV (cP)	CSV (cP)	*ΔH*_*g*_ (J/g)	T_0_ (°C)	T_P_ (°C)	T_C_ (°C)
LN													
WT	212	80.4	488	1500	1465	2522	35	1022	1057	4.16	69.7	68.9	81.2
OX1	188	75.6	400	2650	1418	2631	1232	−19	1213	3.03	62.4	76.2	85.8
OX2	184	74.8	400	2671	1204	2378	1367	−193	1174	3.05	61.9	76.7	83.3
OX3	180	74	396	2558	1236	2606	1322	48	1370	2.99	61.1	76	84.2
S.T.	***	***	***	***	*	ns	***	***	**	***	***	***	**
MN													
WT	196	77.2	456	1745	1722	2837	23	1092	1115	7.12	69.3	69.5	82.3
OX1	172	72.4	396	2834	1412	2693	1422	−141	1281	6.32	60.3	75.2	83.1
OX2	176	73.2	412	2864	1663	2844	1201	−20	1181	6.56	60.2	75.3	84.6
OX3	180	74	404	2946	1655	2936	1291	−10	1281	6.23	60	75.1	84.6
S.T.	***	***	***	***	ns	ns	***	***	**	***	***	***	**

Asterisks denote significant differences between WT and Nhd1‐OX lines at: ns, *P* ≥ 0.05; *, *P* < 0.05; **, *P* < 0.01; ***, *P* < 0.001.

BDV, breakdown viscosity (BDV = PKV−HPV); cP, centipoises; CPV, cool paste viscosity; CSV, consistence viscosity (CSV = CPV−HPV); HPV, hot paste viscosity; PaT, pasting temperature; Pat, pasting time; PKT, peak time; PKV, peak viscosity; S.T., significance test; SBV, setback viscosity (SBV = CPV−PKV); T_o_, T_p_ and T_c_ represent the onset, peak and conclusion gelatinization temperatures, respectively; *ΔH*_g_, the gelatinization enthalpy of starch.

Protein composition in grain usually negatively impacts rice cooking and gelatinization properties (Martin and Fitzgerald, 2002; Nakamura *et al*., 2016). Indeed, enhancing *Nhd1* expression reduced total protein content in grain under both LN and MN supplies (Figure 4g). Glutelin and prolamin are two major components of grain storage protein in rice (Yamagata *et al*., 1982). Accordingly, both glutelin and prolamin concentrations declined due to the enhanced expression of *Nhd1*, although the ratio of glutelin to prolamin had no significant change between WT and Nhd1‐OX lines (Figure S6a). Consistent with the decreased protein content, the grains of Nhd1‐OX lines also contained lower amino acids than WT except for Thr under LN supply (Figure S6b).

Taking together, the better viscosity characteristics and lower protein content in grains confirm that Nhd1 over‐expression remarkably improves rice ECQ irrespective of N supplies.

### Enhancing expression of Nhd1 coordinates the metabolism of carbohydrates and amino acids

**Figure 5:**
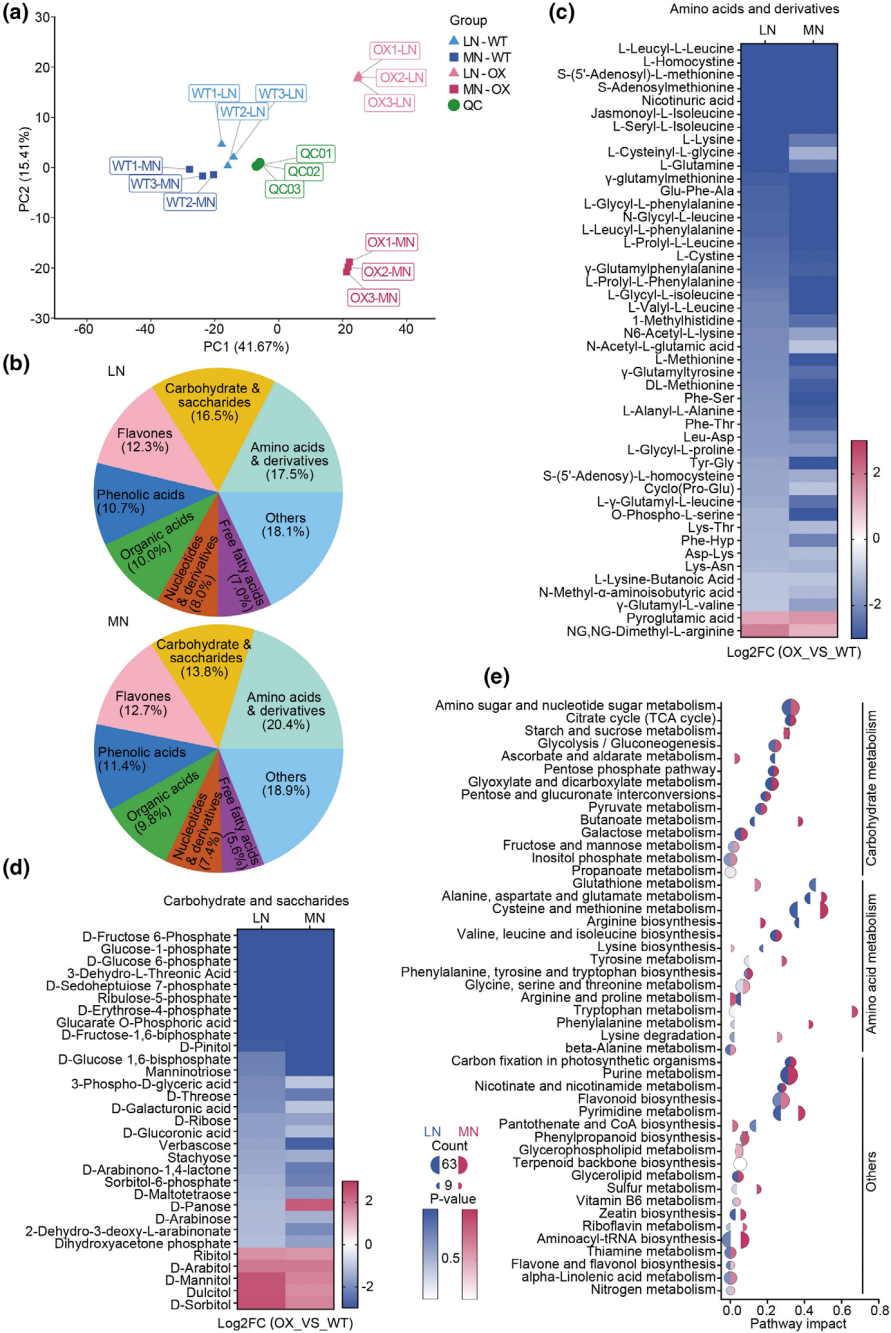
Over‐expression of Nhd1 regulates carbohydrate and amino acid metabolism in grains. (a) Principal component analysis (PCA) of the rice grain metabolism under different N treatments. QCs are standard samples. (b) Classification of changed metabolites by overexpression of Nhd1 under two N treatments. Categories accounted for more than 5% are listed. (c, d) Accumulation difference of metabolites related to amino acids & derivatives clusters (c) and carbohydrate & saccharides clusters (d) between Nhd1‐OX and WT under two N treatments. (e) Function enrichment of metabolites in (c) and (d). Semicircles coloured with red and blue represent different N treatment. Its size shows the count of metabolites.

Grain quality highly relies on appropriate coordination of carbohydrate and N metabolism. To better uncover the mechanism underlying the improvement of grain ECQ in Nhd1‐OX plants, the global metabolic profile in grain was assayed by a widely targeted metabolome analysis. A total of 1012 metabolites were identified, and more than 150 of which were significantly changed by enhanced *Nhd1* expression (Table S2). PCA analysis showed that metabolism profile of Nhd1‐OX plants was clearly separated from WT (Figure 5a). The altered metabolites were mainly assembled in carbohydrate/saccharides cluster (16.45% in LN and 13.76% in MN) and amino acids/derivatives cluster (17.49% in LN and 20.37% in MN) (Figure 5b, Table S3). Interestingly, the abundance of most changed metabolites belonging to these two clusters was significantly reduced in Nhd1‐OX lines compared with that in WT (Figure 5c,d, Table S3). By contrast, some sugar alcohols, such as sorbitol, mannitol and arabitol, were increased in Nhd1‐OX lines (Figure 5d). Since sugar alcohol is reduced‐calorie sweetener and poorly absorbed into the bloodstream (Craig, 2013), Nhd1‐OX rice may be suitable for the diet of diabetics to reduce the change of blood sugar. KEGG pathway enrichment analysis further confirms the biologic processes with regarding to carbohydrate and amino acid metabolisms were the major targets of elevated *Nhd1* during grain ripening which was closely related to the improvement of grain ECQ (Figure 5e, Table S4).

### Nhd1 controls transcriptional profile of ECQ‐related processes

**Figure 6:**
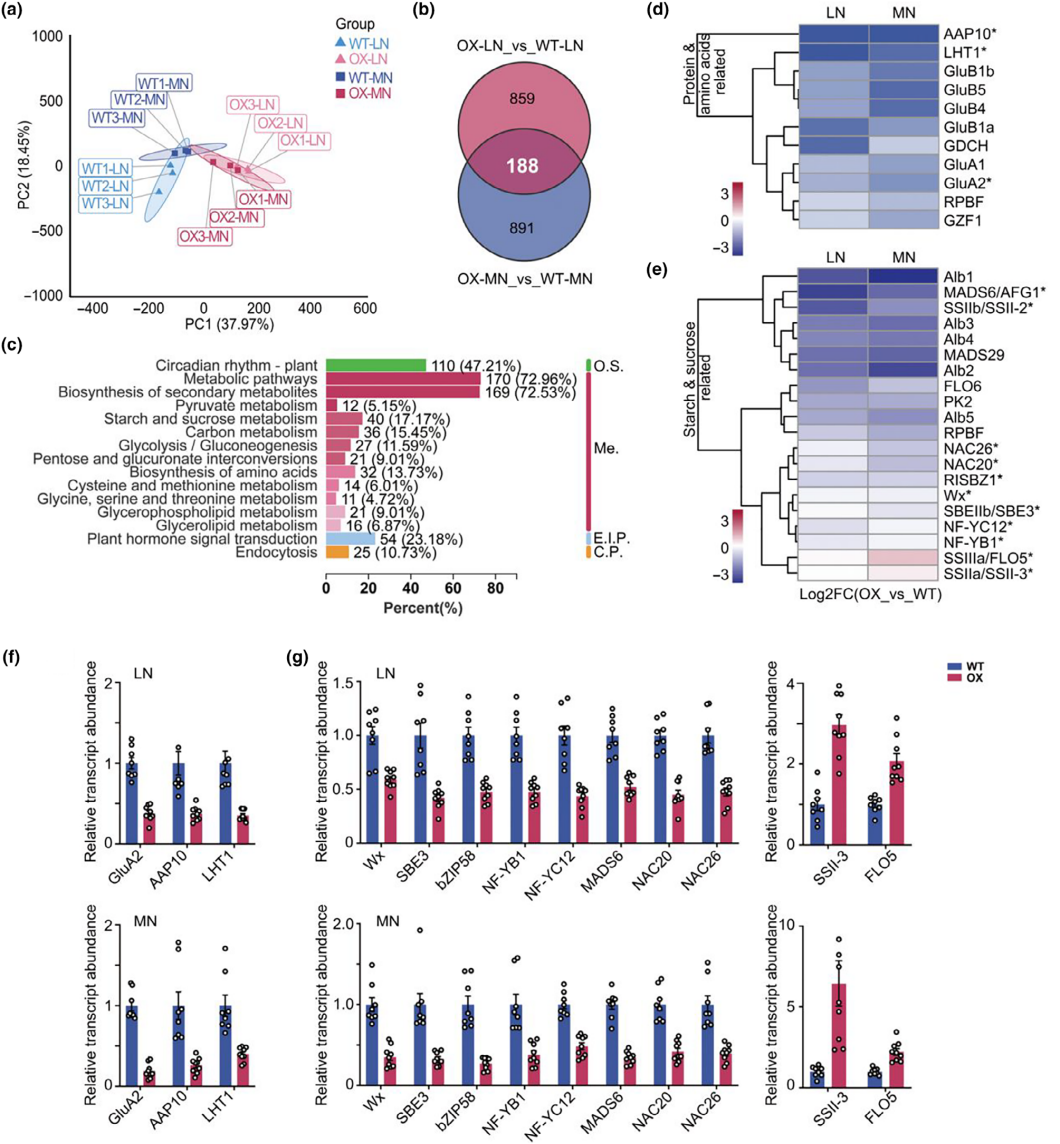
Regulation of Nhd1 on transcription of carbohydrate and amino acid processes. (a) Principal component analysis (PCA) of transcriptomes in the panicle of WT and Nhd1‐OX plants under two different N treatments. (b) Venn diagram of differentially expressed genes (DEGs) affected by overexpression of *Nhd1*. (c) KEGG annotation of DEGs (188 genes shown in b) assigned to the biological process (BP) category (Top 15) under two different N treatment. O.S., organismal system; Me, metabolism; E.I.P., environmental information processing; C.P., cellular processes. (d, e) Expression heatmap of DEGs related to protein and amino acids (d) and starch and sucrose (e) in LN and MN condition. (f, g) qRT‐PCR confirmation of DEGs related to amino acids and derivatives (f) and carbohydrate and saccharides (g) in different N conditions. Enrichment of each gene in transcriptome analysis was marked with asterisks in (d) and (e). The relative abundance was calculated by the 2^−ΔΔCT^ method, and the expression level of each gene in WT was used as the control for which in Nhd1‐OX. Bars are means ± SE, and each point shows a specific value of each plant. One‐tailed Student's *t*‐test was used for the statistical analysis.

To identify the putative genes responsible for the ECQ improvement due to enhancing expression of *Nhd1*, we performed RNA‐seq analysis of panicle sampled at milk period, the critical period of grain establishment (Table S5). PCA analysis showed that transcriptional profile of Nhd1‐OX plants was clearly separated from WT (Figure 6a), indicating the main transcriptional diversity is formed by heredity rather than environment. After gene filtering process, 188 differentially expressed genes (DEGs) between Nhd1‐OX lines and WT were identified (Figure 6b, Table S6). KEGG enrichment analysis of these DEGs was conducted to uncover the key biological processes that are differently regulated by enhancing expression of *Nhd1* (Table S7). It showed that the top three biological terms refer to circadian rhythm pathway, metabolic pathways and biosynthesis of secondary metabolisms pathway, which represent the typical functions of Nhd1 in rice (Figure 6c, Li *et al*., 2022a,b; Wang *et al*., 2020; Zhang *et al*., 2021d). Besides, many carbohydrates and amino acid metabolic pathways that are closely relative to ECQ regulation were also identified among these 188 DEGs (Figure 6c, red column).

Over‐expression of *Nhd1* significantly altered expression of genes related to starch and sucrose metabolism (Figure 6e, Table S9). Key genes in starch synthesis process, including *Wx* that encodes granule‐bound starch synthase I (*GBSSI*) (Wang *et al*., 1990) was downregulated by over‐expression of *Nhd1* regardless of N supply (Figure 6g). Several other known transcription factors in the starch synthesis pathway were also reduced in Nhd1‐OX lines (Figure 6g). The expression pattern of these tested genes explained the decline of amylose in Nhd1‐OX lines. Meanwhile, the expression of *SSII‐3* and *FLO5* that are contributing to amylopectin formation (Tian *et al*., 2009) were markedly upregulated by over‐expression of *Nhd1* (Figure 6g), coinciding with a raise of GC in the grain of Nhd1‐OX lines (Figure 4b). Therefore, data from transcriptome and gene expression analysis elucidates that multiplying the expression of *Nhd1* mainly acts on carbohydrate and amino acids metabolisms to improve grain ECQ in rice.

Several genes directly participated in protein and amino acid metabolism were also substantially downregulated in Nhd1‐OX lines compared with WT (Figure 6d, Table S8). RT‐qPCR analysis confirmed that over‐expression of *Nhd1* reduced the transcript levels of amino acid transporters that are responsible for N remobilization during grain filling, such as *amino acid permease 10* (*OsAAP10*), *Lysine‐Histidine‐type Transporter 1*(*OsLHT1*) and the expression of glutelin *OsGlutelin type‐A (GluA2)* (Figure 6f), thus leading to the lower amino acid and protein accumulation in the grains of Nhd1‐OX lines (Figures 4g and 5c, S6).

As a MYB transcription factor, Nhd1 regulates the expression of its target genes via binding two conserved cis‐acting elements, NBS (Nhd1‐binding site; Zhang *et al*., 2021d) and EE (evening element; Lee *et al*., 2022). Interestingly, cis‐acting element analysis identified totally 67 NBS and 54 EE elements existed in the 2000 bp promoter regions of the 188 DEGs in Nhd1‐OX lines (Figure S7a, Table S10). 42 out of 188 DEGs (~22%) have at least one Nhd1 binding elements in their promoter regions (Figure S7b). Key genes involved in carbohydrate and amino acids metabolism processes and regulated by the over‐expression of *Nhd1*, such as *LHT1*, *GluA2*, *bZIP58*, *NAC26* and *SSII‐3*, have multiple NBS or EE elements in their promoter sequences (Figure S7c). Moreover, EMSA assay demonstrated that Nhd1 can directly bind to both NBS and EE in promoter of Os*LHT1* and *GluA2*, and only binds to NBS motifs in promoter of *SSII‐3* in vitro (Figure S7d). These results further indicate that Nhd1 is likely to play a direct role in regulating grain ECQ via targeting key genes for carbohydrate and amino acid metabolisms.

### Genetic association of better pasting characteristic with higher expression level of *Nhd1* in rice cultivars

To evaluate the genetic association of Nhd1 with grain ECQ in rice, we tested the expression levels of *Nhd1* and pasting characteristics in 22 main rice cultivars belonging to *japonica* subspecies for current rice production in Jiangsu province of China under LN (150 kg N/ha) and MN (250 kg N/ha) supplies. Even though these tested rice cultivars are grown in the same province nowadays, the expression levels of *Nhd1* and pasting characteristics varied largely in our field trial (Figure S8a,b). Regardless of their genetic background, higher N treatment remarkably induced the expression of *Nhd1*, whereas the pasting parameters exhibited fewer changes in response to altered N supplies (Figures S8a,b and S9a), implying that the genetic diversity across the testing rice cultivars rather than the environment factor is the major determinant of grain ECQ. Interestingly, we found that the cooking and gelatinization properties of grains were significantly correlated with the expression level of *Nhd1* among these 22 rice cultivars (Figure S8c). Specifically, the pasting temperature and breakdown viscosity were negatively correlated with transcript abundance of *Nhd1* irrespectively of N applications, while the peak viscosity and setback viscosity were positively correlated with the expression level of *Nhd1* (Figures S8c and S9b).

## Discussion

**Figure 7:**
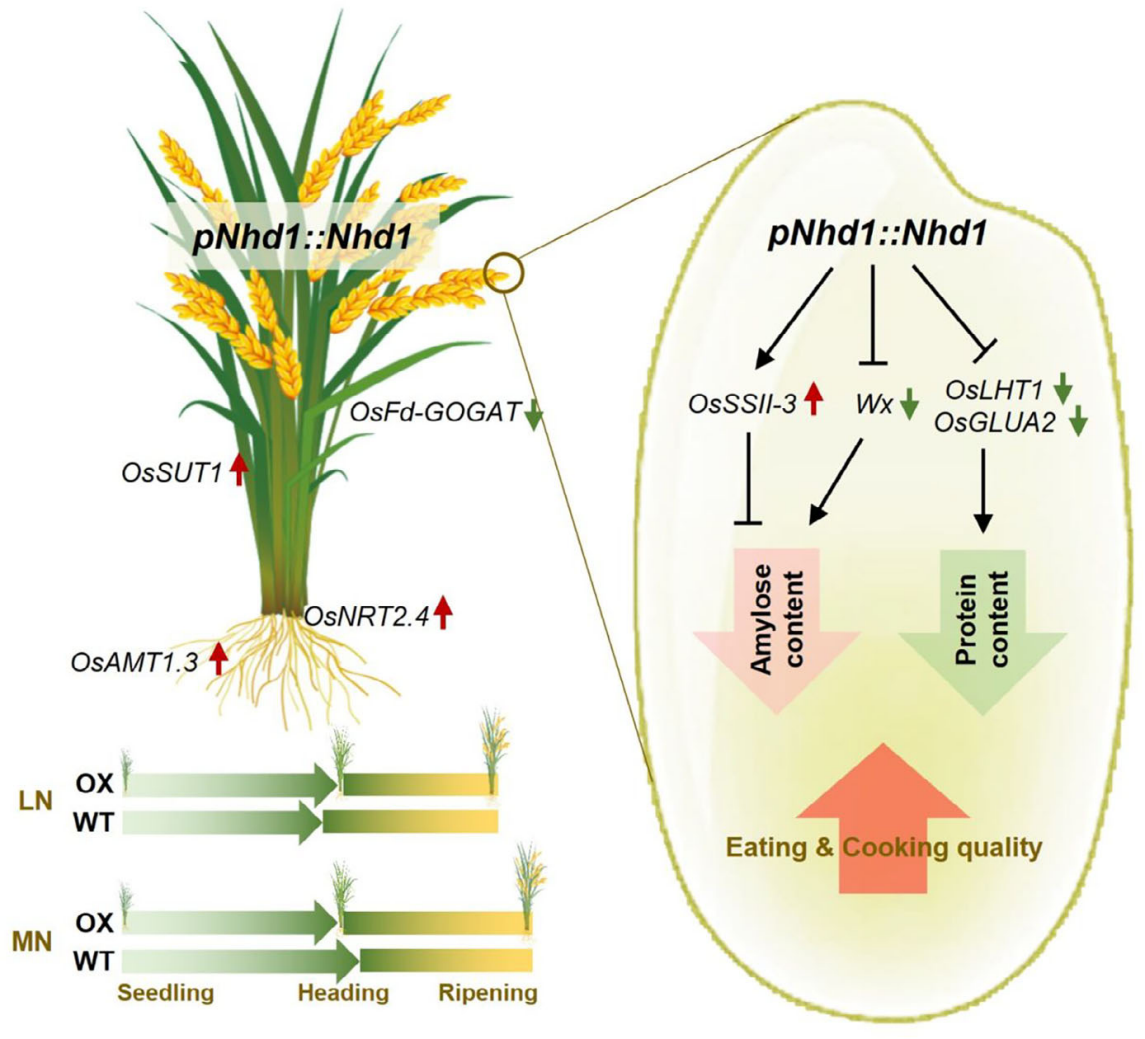
Working model of Nhd1‐mediated grain ECQ improvement in rice. Native promoter‐controlled enhancement of Nhd1 expression in rice reduces the sensitivity of heading date to different N supplies without altering the entire length of growth duration or grain yield. Meanwhile, increased expression of Nhd1 regulates key genes involved in carbohydrate and amino acid metabolisms to decrease amylose and protein content in grains, which significantly improves grain ECQ in rice. Therefore, multiplying *Nhd1* expression under the control of its native promoter has been proved as a practical strategy to upgrade grain ECQ for rice production.

Nhd1 as a key circadian regulator has pleiotropic functions on heading date, plant growth, stress tolerance and NUE in rice (Li *et al*., 2022a,b; Sun *et al*., 2021; Wang *et al*., 2020; Zhang *et al*., 2021d). Here, we report that enhancing endogenous expression of *Nhd1* driven by its native promoter leads to a change of key genes involved in metabolisms of carbohydrates and amino acids to reduce content of both amylose and protein in grains, thus significantly upgrading the rice ECQ irrespective of N supply levels (Figure 7). Remarkably, the Nhd1 controlled ECQ improvement neither affects grain yield nor NUE (Figures 2 and 3). Interestingly, starch pasting properties in local 22 main rice cultivars are positively correlated with the transcript abundance of *Nhd1* (Figure S8). The results suggest that the expression level of *Nhd1* can be used as a potential breeding target to improve rice grain ECQ.

Starch and protein in rice grain are two major factors affecting ECQ (Chang *et al*., 2009; Nakamura *et al*., 2016). Generally, the grain with lower AC and GT but higher GC is relatively softer and stickier after cooking (Han and Hamaker, 2001; He *et al*., 2010; Peng *et al*., 2021). We showed that enhanced expression of Nhd1 led to an increase of the gel consistency (GC), but a decrease of onset gelatinization temperature (GT) and pasting temperature, following a reduce of amylose content (AC) (Figure 4a–d; Table 1). Previous study demonstrated two central genes, *Wx* and *SSII‐3*, which are critical for influencing starch properties and ECQ in rice (Tian *et al*., 2009). Specifically, reducing abundance of *Wx* results in softer rice with lower AC, while enhancing expression of *SSII‐3* improves ECQ with lower AC but higher GC (Tian *et al*., 2009; Zhang *et al*., 2021a). The over‐expression of *Nhd1* dramatically inhibited expression of *Wx* but induced expression of *SSII‐3*, particularly at relative high N condition (Figure 6e,g), thus contributing to an improvement of grain ECQ.

Protein accumulation in grains relies on the coherence of amino acid translocation from source tissues via amino acid transporters and protein synthesis (He *et al*., 2021; Tegeder and Masclaux‐Daubresse, 2018). In rice, OsLHT1 is a major amino acid transporter for N re‐allocation to seeds (Guo *et al*., 2020) and grain protein content is highly positively associated with transcription level of glutelin synthesis gene *GluA2* (Shewry *et al*., 1995; Yang *et al*., 2019). Interestingly highly expression of *Nhd1* inhibited expression of both *LHT1* and *GluA2* (Figure 6d,f), which partially explains the decreased accumulation of amino acid metabolites in Nhd1‐OX grains (Figure 5d). Furthermore, the regulation of Nhd1 on starch and protein synthesis is validated by alterations both in metabolite profiles and transcription levels related to carbohydrates and amino acid metabolism in Nhd1‐OX lines (Figures 5e and 6c). In addition, low AC content leads to decrease of resistant starch ratio in Nhd1‐OX grain (Figure 4c), indicating its higher digestibility and glycemic index (GI) (Huang *et al*., 2023; Kendall *et al*., 2004). On the other hand, despite the unchanged ratio of glutelin to prolamin, glutelin content decreased due to the overall decline in total protein content in Nhd1‐OX grains (Figure S6a). Thus, for individuals with kidney diseases, Nhd1‐OX rice with lower glutelin content could benefit for alleviating the metabolic burden by restricting protein intake (Obi *et al*., 2018).

It is worth to be noted that the influence of Nhd1 on heading date may also contribute to the improvement of rice ECQ. We observed in our field trials that over‐expression of *Nhd1* altered heading date but not mature time at the same N condition, implying the different grain filling period of Nhd1 over‐expression lines and WT (Figure 1). Limited N supply can promote filling rate but reduce its stage duration which in turn deteriorate grain quality with high AC (Zhang *et al*., 2021b; Zhao *et al*., 2022). Indeed, we detected the similar results in WT (Figures 1e and 4a). Even though the grain filling duration of Nhd1‐OX plant was shortened in LN condition, the limited preparation of material and enzyme for amylose synthesis may repair the damage of ECQ by decreasing AC in Nhd1‐OX grain (Figure 4a). In contrast, due to the earlier heading, Nhd1‐OX plant had an extended grain filling period in MN condition (Figure 1e) as well as better properties of starch and protein, which contributed to the increase of ECQ (Figure 4; Table 1).

Previously, Nhd1 has been clarified as a central player in the transcriptional regulation of circadian rhythm process (Figure 6c; Ogiso *et al*., 2010; Sun *et al*., 2021; Zhang *et al*., 2021d; Zhang *et al*., 2022). Physiological processes, such as starch and amino acid metabolisms, highly rely on photosynthesis during the daytime and respiration during the night, thus, which are regulated by day/night rhythm (Bellet *et al*., 2011; Farré and Weise, 2012; Graf and Smith, 2011; Harmer *et al*., 2000; Li *et al*., 2016). It has been demonstrated that more than 30% of genes transcript abundance and 30% of primary metabolite accumulation in *Arabidopsis* are governed by circadian rhythm (Covington *et al*., 2008; Harmer *et al*., 2000; Li *et al*., 2016). Interestingly, oscillation expression of *GBSSI* (homologue of *Wx*) and *SBE* at day and night converses in many species (Mérida *et al*., 1999; Mutisya *et al*., 2009a,b; Pao *et al*., 2005; Ral *et al*., 2006; Tenorio *et al*., 2003; Wang *et al*., 2001, 2004). Both Nhd1 in rice and its homologue CCA1 in *Arabidopsis* have been identified to regulate gene's transcription by targeting two conserved cis‐acting elements, NBS (Nhd1‐binding site in rice, also called CBS in *Arabidopsis*; Zhang *et al*., 2021d) and EE elements (Lee *et al*., 2022). CCA1 can directly regulate *GBSSI* by binding the CBS element in *Arabidopsis* (Tenorio *et al*., 2003). In rice, we did not find any NBS or EE motif in the promoter region of *Wx*. However, there were two NBS motifs in *SSII‐3* promoter and one NBS in *SSIIIa* (*Flo5*) promoter, implying a direct regulation of clock gene Nhd1 on starch synthesis in rice (Figures 6e,g and S7). The metabolome results showed that many metabolites involved in C and N metabolism were strongly affected by over‐expression of *Nhd1* (Figure 5). The abundance of metabolites functioning in glucose metabolism, such as fructose, fructose 6‐P, glucose‐1‐P, glucose 6‐P and glucose 1,6‐P2, were decreased in Nhd1‐OX plant (Figure 5d), while a similar result was detected in CCA1 over‐expression lines of *Arabidopsis* (Fukushima *et al*., 2009). Glucose metabolism has been found to be tightly linked with circadian rhythm both in animal and plant (Ch *et al*., 2020; Kim *et al*., 2017), suggesting the Nhd1 regulation of this process in the circadian clock module. The product in downstream of these metabolites, ADP‐glucose, is imported into plastid for starch synthesis (Ohdan *et al*., 2006; Zhou *et al*., 2016). Thus, over‐expression of Nhd1 reduced the materials for starch synthesis that coordinated with inhibition of *Wx* and induction of *SSIII‐3* to repress synthesis of amylose and promote amylopectin chain elongation (Figures 6e,g and 7). In contrast, the accumulation of glutamine, which is a key transit compound in amino acid metabolism, decreased in Nhd1‐OX rice (Figure 5b) but increased in CCA1‐ox *Arabidopsis* (Fukushima *et al*., 2009). It had been demonstrated that CCA1 promotes glutamine synthesis by inducing expression of *GLN1.3* in *Arabidopsis* (Gutiérrez *et al*., 2008). However, we identified that Nhd1 negative controls glutamine synthesis via inhibiting expression of *OsFd‐GOGAT* and *OsGS1.1* (Figure 3f; Li *et al*., 2022a). Distinct regulations on N assimilation between rice and *Arabidopsis* likely cause the different responses of glutamine abundance to over‐expression of Nhd1 or CCA1.

In general, most genes' functions rely on their unique expression patterns in specific tissues or in certain manner responding to environmental conditions (Ma and Bohnert, 2007; Porto *et al*., 2014). Fluctuation of *Nhd1* expression follows a circadian rhythm in rice (Wang *et al*., 2020; Zhang *et al*., 2021d). Using a native promoter to multiply the abundance of *Nhd1* allowed us to enhance its expression intensity without altering its original expression rhythm (Figure S1e). By contrast, when increasing *Nhd1* transcription by a CaMV35S promoter, its expression peak has no significant change but its expression rhythm is disturbed in constant dark condition (Wang *et al*., 2020). Therefore, these two strategies to enhance *Nhd1* expression were supposed to generate different phenotypes. Indeed, the tiller number and yield were unchanged in Nhd1‐OX plant by using a native promoter (*pNhd1::Nhd1*) (Figure 2b,e) whereas these two traits were remarkably decreased in Nhd1‐OX lines by using a constitutive expressing promoter (*p35S::OsCCA1*) (Wang *et al*., 2020), although the early heading phenotype was observed in both strategies. It is likely that the constitutive expressing driven by 35S promoter disrupted the original expression specificity of *Nhd1*, which may be the main reason to cause a distinct phenotype compared with that by using a native promoter. In rice, *Nhd1* is highly expressed in leave (special in flag leaf) and inflorescences but its transcript abundance is much lower in node and internode in reproductive stage (Lee *et al*., 2022; Wang *et al*., 2020; Zhang *et al*., 2021d). Compared with the native promoter lines, *Nhd1* constitutive expression increased *Nhd1* transcript abundance much stronger in the node and internode, thus leading to a much stronger inhibition of tillering (Wang *et al*., 2020). Similarly, it has been reported that compared with the delayed flowering time displaying in constitutive expression of *CCA1* plant, guard cell‐specific induction of *CCA1* is no longer affecting flowering time in *Arabidopsis* (Hassidim *et al*., 2017). These results confirm that maintaining the original expression pattern and rhythm is critical for the proper function of Nhd1 in rice or its homologue CCA1 in *Arabidopsis*. In this context, enhancing *Nhd1* expression level by using its native promoter rather than a constitutive promoter is a more promising strategy to avoid unexpected or artificial side‐effects in rice.

## Experimental procedures

### Construction of transgenic rice

2500 bp sequence upstream of transcriptional start site (ATG) was used as *Nhd1* promoter sequence. CDS of Nhd1 promoted by its native promoter fusing with GUS were cloned into pCE2 TA/Blunt‐Zero vector (Lot. #R601, Vazyme Bio, Nanjing, China). The constructed *pNhd1::Nhd1* was shown in Figure S1a. The Nhd1‐OX lines was generated via Agrobacterium transgenic system in *O. sativa japonica* cv. Nipponbare background (Chen *et al*., 2017). The primers used for this study are reported in Table S1.

### Growth conditions and phenotype measurement

The Nhd1‐OX and WT plants were cultivated in field plots at the Experimental Station of Nanjing Agricultural University with a subtropical climate from May to October in a year. The seed sterilization and seedling preparation in paddy field nursery of Nhd1‐OX lines and WT were the same as previously reported (Chen *et al*., 2017; Zhang *et al*., 2021d). The seedlings with near the same size were transplanted to field plots after 3 weeks of germination. WT and Nhd1‐OX lines were closely planted in plots supplied with 150 kg N/ha for LN treatment or 250 kg N/ha for MN treatment. Each plot was 2 × 2.5 m in size and the seedlings were planted in 10 × 10 arrays. Plants at the edges of all four sides of each plot were removed at maturity to avoid the influence of edge effects.

Various agronomic traits including days to heading (DTH), days to ripening (DTR), panicle number, grain number per panicle and thousand grains weight (1000‐grain weight), grain yield, panicle weight, secondary branches number, seed setting rate, were manually measured. DTH was counted from seed transplanting to first panicle heading about 1–2 cm of each plant. DTR was counted from seed transplanting to over 95% of the panicle turns yellow of each plant. The reproductive tillers having panicles with filled grains were counted for panicle number. Thousand grain weight was determined by measuring the weight of harvested seeds that were air‐dried until they reached ~14.0% moisture content. Panicle‐related traits were measured from main tillers of over five plants in WT and Nhd1‐OX lines, respectively.

### RNA isolation and gene expression analysis

Total RNAs were prepared from the various tissues of the WT and transgenic plants using Trizol reagent (Lot. #15596018, Thermo Fisher Scientific, MA). First‐strand cDNA was synthesized using a HiScript III 1st Strand cDNA Synthesis Kit (Lot. #R312, Vazyme Bio, Nanjing, China). The expression levels were measured by quantitative reverse transcription‐PCR (qPCR) using the ABI QuabtStudio6 Flex real‐time PCR system (Thermo Fisher Scientific, MA). The *OsActin1* gene (LOC_Os03g50885) was used as the internal control. At least three replicates were performed for each analysis. Relative expression levels of the examined genes were determined according to the 2^−ΔCT^ and 2^−ΔΔCT^ methods (Livak and Schmittgen, 2001).

### Southern blot analysis

The independent transgenic lines with over‐expression of Nhd1, namely OX1, OX2 and OX3, were determined by Southern‐blot analysis. Genomic DNA was extracted from leaves of WT and T1 transgenic plants using the SDS method, and 8 μg of genomic DNA was digested with the restriction enzyme HindIII and EcoRI overnight at 37 °C. The digested DNA was separated on a 0.8% (w/v) agarose gel, transferred to a Hybond‐N+ nylon membrane and hybridized with the coding sequence of the hygromycin‐resistant gene used as the hybridization probe following the procedures described previously (Murray and Thompson, 1980).

### Western blot

The Western blot process was described by Chen *et al*. (2017). The total protein of 10 g shoots was sampled and 50 μg of each protein was analysed in gel‐loaded buffer and boiled in 10% SDS‐PAGE. Protein transfer to PVDF membrane and incubated with OsActin (1: 5000) or Nhd1 (1: 2000) overnight at 4 °C. The membrane was then incubated with the appropriate secondary antibody (1: 20000; Pierce), then carries on the chemiluminescence detection.

### Electrophoretic mobility shift assay (EMSA) assay

Full length CDS of Nhd1 was cloned into pET29a (+) vector (Novagen) which was transformed into Rosetta 2 strain of Escherichia coli to expression His‐Nhd1 fusion protein (Zhang *et al*., 2021d). This recombinant protein was purified using Ni‐NTA agarose (Sangon Biotech, Shaihai, China). The binding activity of the proteins was analysed using an oligo nucleotide containing EE‐ or NBS‐ motif labelled both at 3′ and 5′ end (Genscript, Nanjing, China). The protein‐probe mixture contained binding buffer (25 μL) were used for gel electrophoresis. And assay was performed as described by Zhang *et al*. (2021d).

### Quantification of plant N content and N use efficiency (NUE)

The entire or separated tissues of plants were first dried and weighted, and then the representative dry samples after being ground were digested following the Kjeldahl's method (using H_2_SO_4_‐H_2_O_2_). The concentration of N in the digested solution after dilution was measured using Autoanalyzer AA3 (Seal, German). NUE, NUpE and NUtE were calculated according to the method of Xu *et al*. (2012).

### Analysis of grain ECQ properties

The mature seeds were first dried in a drying oven (40 °C) before milling to white rice. A fraction of the milled rice was ground into flour and used for starch isolation after passing through a 100‐mesh aperture, as described by Zhang *et al*. (2013). Amylose content (AC) was determined by the iodine colorimetric method (ISO 6647‐1:2007) using Epoch Microplate Spectrophotometer (BioTek, Winooski, Vermont, United States) according to the protocol described by Tang *et al*. (2002). Amylose content values were calculated from a standard curve established using mixture solutions of amylose and amylopectin. Gel consistency (GC) was measured according to the method of Cagampang *et al*. (1973). N concentration of the milled rice was determined using an Elemental Analyzer (Elementar, Germany), and then converted into protein content (PC) using a conversion factor (5.95). Amino acid content in the milled rice was determined using LA8080 (HITACHI ltd., Japan) according to the method of Wang *et al*. (2007).

For RVA analysis, a rapid viscosity analyser (RVA4500) (Perten, Sweden) and analyse software TCW3 (Perten, Sverige) were used. The operation process was based on the American Association of Cereal Chemists (AACC) operating procedure. RVA refers to viscosity change of rice starch during heating. Indicators of starch gelatinization were analysed including peak viscosity, hold viscosity, final viscosity, breakdown, setback, peak time and pasting temperature. Thermal properties were measured according to the method of Zhang *et al*. (2013) and investigated by differential scanning calorimetry (DSC) (Model 200 F3 Maia, Netzsch Instruments, Bavaria, Germany). Retrogradation percentage (%R) was calculated as %R = ΔH_rep_/ΔH_gal_ × 100.

For SEM observations of the cross‐cut rice grains, samples were directly mounted on an aluminium stub using carbon double‐sided conductive tape. The samples were then observed and photographed after being coated with gold using a sputter coater and examined using an environmental scanning electron microscope (Apreo 2, Thermo Fisher Scientific, MA). The resistant starch (RS) content and total digestible starch content were determined by a digestible and resistant starch assay kit (K‐DSTRS, Megazyme, Ireland) according to the method of Huang *et al*. (2020a). The glutelin and prolamin extraction were performed as described by Takemoto *et al*. (2002), and glutelin and prolamin content were analysed by SDS–PAGE (Udaka *et al*., 2000; Wang *et al*., 2010).

### Metabolome analysis

Rice seed of wild‐type and Nhd1‐OX were collected from moderate‐ and low‐nitrogen fields. Samples were freeze‐dried by vacuum freeze‐dryer (Scientz‐100F). The freeze‐dried sample was crushed using a mixer mill (MM 400, Retsch) with a zirconia bead for 1.5 min at 30 Hz. Samples extraction and preparation before UPLC–MS/MS analysis accord to the method of Chen *et al*. (2013). UPLC and ESI‐Q TRAP–MS/MS were made by Metware Biotech. Co., Ltd (Wuhan, China). Data analysis and graphing were analysed by Metware Cloud, a free platform (https://cloud.metware.cn). KEGG pathway was plotted using OmicStudioKits (v. 1.8.1) online tool at https://www.omicstudio.cn/tool.

### RNA‐seq analysis

Young panicle of WT and Nhd1‐OX plants were collected. RNA extraction was used RNeasy Micro Kit (Lot.74004, Qiagen, Germany). The cDNA libraries were sequenced on the Illumina sequencing platform by Metware Biotechnology Co., Ltd. (Wuhan, China). Clean reads were compared to oryza_sativa_Ensembl_52 (ftp://ftp.ensemblgenomes.org/pub/plants/release‐52/fasta/oryza_sativa/dna/). Data analysis and graphing were analysed by Metware Cloud, a free platform (https://cloud.metware.cn).

### Statistical analysis

Data were analysed by Tukey's test of one‐way analysis of variance (ANOVA). *P*‐value on the histograms indicate statistically significant differences at *P* < 0.05 between the transgenic plants and WT (one‐way ANOVA), while ns indicate statistically no significant differences at *P* ≥ 0.05. Data are presented as the means ± standard deviation (SD), shown by error bars, except that are presented as the means ± standard error (SE) in the ECQ related gene analysis, also shown by the error bars. All statistical evaluations were conducted using the Microsoft Office Excel version LTSC 2021 software (Microsoft Inc., WA).

## Accession numbers

Genes from this article can be found on the EnsemblPlants website (http://plants.ensembl.org/index.html) under the following accession numbers: *Nhd1* (Os08g0157600), *OsGI* (Os01g0182600), *SPL16* (Os08g0531600), *GW7* (Os07g0603300), *OsAMT1;3* (Os02g0620500), *OsNRT2.4* (Os01g0547600), *Fd‐GOGAT* (Os07g0658400), *AAP10* (Os02g0722400), *LHT1* (Os08g0127100), *GluB1b* (Os02g0249900), *GluB5* (Os02g0268100), *GluB4* (Os02g0268300), *GluB1a* (Os02g0249800), *GDCH* (Os10g0516100), *GluA1* (Os01g0762500), *GluA2* (Os10g0400200), *RPBF* (Os02g0252400), *GZF1* (Os07g0668600), *Alb1* (Os07g0214100), *MADS6* (Os02g0682200), *SSII*‐*2* (Os02g0744700), *Alb3* (Os07g0213800), *Alb4* (Os07g0214600), *MADS29* (Os02g0170300), *Alb2* (Os07g0214300), *FLO6* (Os03g0686900), *PK2* (Os07g0181000), *Alb5* (Os07g0215500), *SSII*‐*3* (Os06g0229800), *FLO5* (Os08g0191433), *NF*‐*YB1* (Os02g0725900), *NF‐YC12* (Os10g0191900), *SBE3* (Os02g0528200), *Wx* (Os06g0133000), *RISBZ1* (Os07g0182000), *NAC20* (Os01g0104500) and *NAC26* (Os01g0393100).

The raw transcriptome reads have been deposited in the NCBI Sequence Read Archive (SRA) database under accession: PRJNA924570.

## Funding

This study was supported by Jiangsu Provincial Key Research and Development Program (BE2022336, BE2020339), National Natural Science Foundation of China (31930101, 32102474), Natural Science Foundation of Jiangsu (BK20210388), Jiangsu Seed Industry Revitalization Project (JBGS [2021] 011) and Fundamental Research Funds for the Central Universities (KYQN2022022).

## Conflict of interests

The authors declare no competing interest.

## Author Contributions

Y.Z. and S.Z. planned and designed the research, generated Nhd1‐OX lines. Y.Z. conducted most of the experiments. J.Z. and W.W. help to finish the field experiment and nitrogen use efficiency analysis. Y.Z. and T.Z. made bioinformatics analysis of transcriptome and metabolome. Y.Z., S.Z., Y.L. and G.X. made the data analysis, wrote the manuscript. Y.Z. and S.Z. contributed to equally. All authors contributed to the finalization of the manuscript.

## Supporting information


**Figure S1** Generation and characterization of Nhd1‐OX transgenic lines.
**Figure S2** Overexpression of Nhd1 reduces the sensitivity of heading date to different N supplies at the protein level.
**Figure S3** The heading and ripening phenotype of WT and Nhd1‐OX lines under two N treatments in the field trial of second growth season.
**Figure S4** Panicle characteristics of WT and Nhd1‐OX lines under different N supplies.
**Figure S5** Total C and N status in the grains of WT and Nhd1‐OX lines under two N treatments.
**Figure S6** Quantification of grain storage proteins and main amino acids in WT and Nhd1‐OX lines in two different N conditions.
**Figure S7** Nhd1‐binding element analysis on the promoter of Nhd1‐regulated genes.
**Figure S8** Correlation between *Nhd1* transcript abundance and pasting characteristics in local main rice cultivars.
**Figure S9** Improving rice pasting characters by enhancing native promoter‐controlled expression of *Nhd1*.

**Table S1** Primer sequences used in this study.
**Table S2** The relative abundance of 1012 metabolites by wildly targeted metabolome.
**Table S3** Classification and fold change analysis of 1012 metabolites in wildly targeted metabolome.
**Table S4** KEGG‐PATHWAY enrichment analysis for differential metabolites in wildly targeted metabolome.
**Table S5** The relative abundance of transcripts in transcriptome.
**Table S6** Function information and differential expression analysis of transcripts in transcriptome.
**Table S7** Classification and functional process of differential expression genes affected by overexpression Nhd1.
**Table S8** The relative abundance and fold change analysis of protein & amino acids related genes in transcriptome.
**Table S9** The relative abundance and fold change analysis of starch & sucrose related genes in transcriptome.
**Table S10** Nhd1 binding sites analysis in differential expression genes affected by overexpression Nhd1.
