# Supplementary material for: Improving rice eating and cooking quality by enhancing endogenous expression of a nitrogen‐dependent floral regulator
Source: Plant Biotechnol J. 2023 Aug 25;21(12):2654–70. doi: 10.1111/pbi.14160 (PMC10651157; doi:10.1111/pbi.14160)
Supplement: Supplementary file 1 — Figure S1 Generation and characterization of Nhd1‐OX transgenic lines. Figure S2 Overexpression of Nhd1 reduces the sensitivity of heading date to different N supplies at the protein level. Figure S3 The heading and ripening phenotype of WT and Nhd1‐OX lines under two N treatments in the field trial of second growth season. Figure S4 Panicle characteristics of WT and Nhd1‐OX lines under different N supplies. Figure S5 Total C and N status in the grains of WT and Nhd1‐OX lines under two N treatments. Figure S6 Quantification of grain storage proteins and main amino acids in WT and Nhd1‐OX lines in two different N conditions. Figure S7 Nhd1‐binding element analysis on the promoter of Nhd1‐regulated genes. Figure S8 Correlation between Nhd1 transcript abundance and pasting characteristics in local main rice cultivars. Figure S9 Improving rice pasting characters by enhancing native promoter‐controlled expression of Nhd1. [file PBI-21-2654-s002.docx]

**Supporting Information**


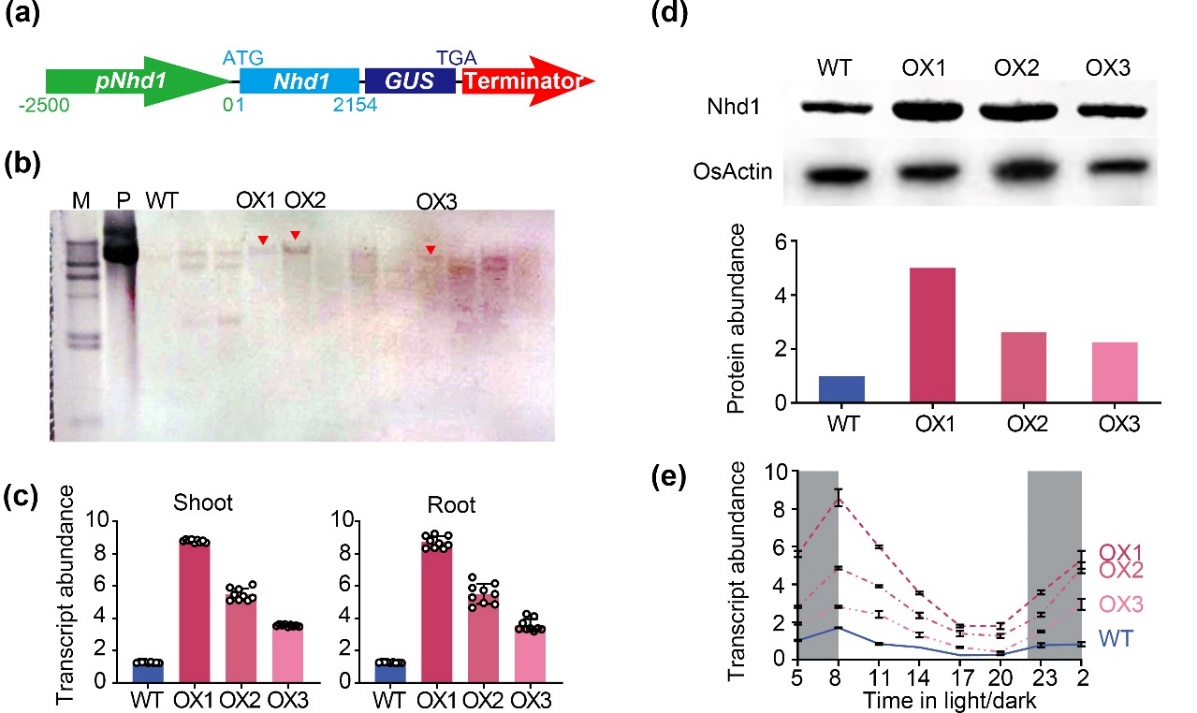


**Figure S1. Generation and characterization of Nhd1-OX transgenic lines.**

(**a**) Construction of *pNhd1::Nhd1* expression vector. (**b**) Southern blot of genomic DNA isolated from Nhd1-OX lines and WT. Hybridization using a hygromycin gene probe. P, positive control, M, marker label. (**c**) Relative transcript abundance of *Nhd1* in shoots and roots of Nhd1-OX lines and WT plants under MN condition. Each circle spot shows a specific value for each biological replicate. (**d**) Western blot of total proteins from shoots of Nhd1-OX lines and WT. Hybridization with an OsActin-specific antibody and an Nhd1-specific antibody. Each line was loaded with equal quantity of protein. (**e**) 24h-circadian of *Nhd1* relative transcript abundance in shoots of WT and Nhd1-OX plants. Relative transcript levels in (c) and (e) were determined by qRT-PCR using the 2^–ΔCT^ method and normalized by using *OsActin1* as internal control. Bars represent means ± SD, n = 6 biological replicates.


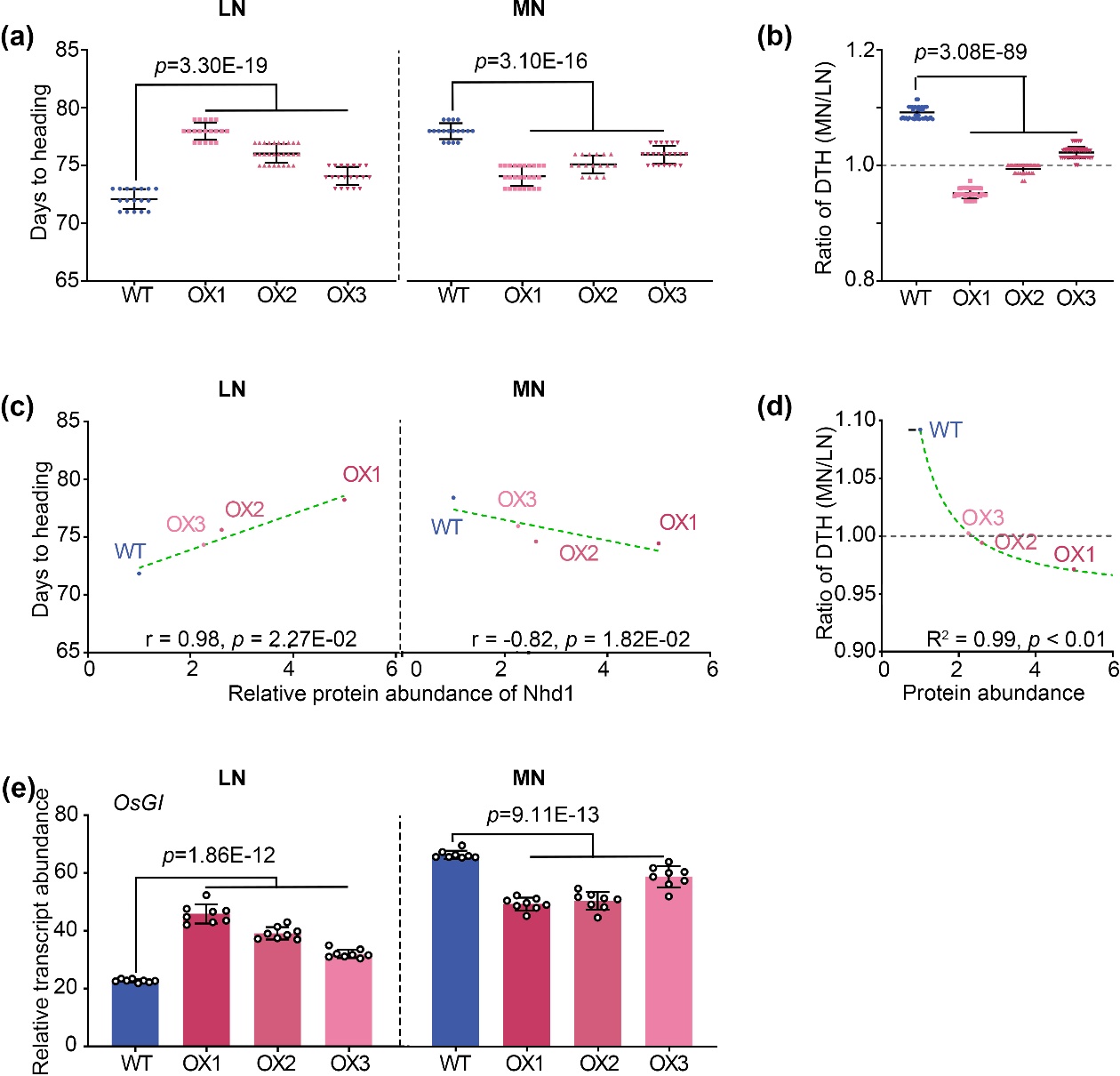


**Figure S2. Overexpression of Nhd1 reduces the sensitivity of heading date to different N supplies at the protein level.**

(**a**) Days to heading (DTH) after transplanting in WT and Nhd1-OX lines under LN and MN treatments. (**b**) The ration of heading date (DTH) between different N treatment. (**c**) The correlations between *Nhd1* protein abundance and DTH. The green line represents the correlation fitting curve, and the p- and r- values of the curve have been given in the graph. (**d**) The correlations between *Nhd1* protein abundance and the ratio of DTH. The green line represents the correlation fitting curve, and the p- and r- values of the curve have been given in the graph. (**e**) The expression of *OsGI* in WT and Nhd1-OX plants under different N treatments. Bars are means ± SD, and the black dash represents the average value of each group while each point shows a specific value of each plant. One-tailed Student’s t-test was used for the statistical analysis.


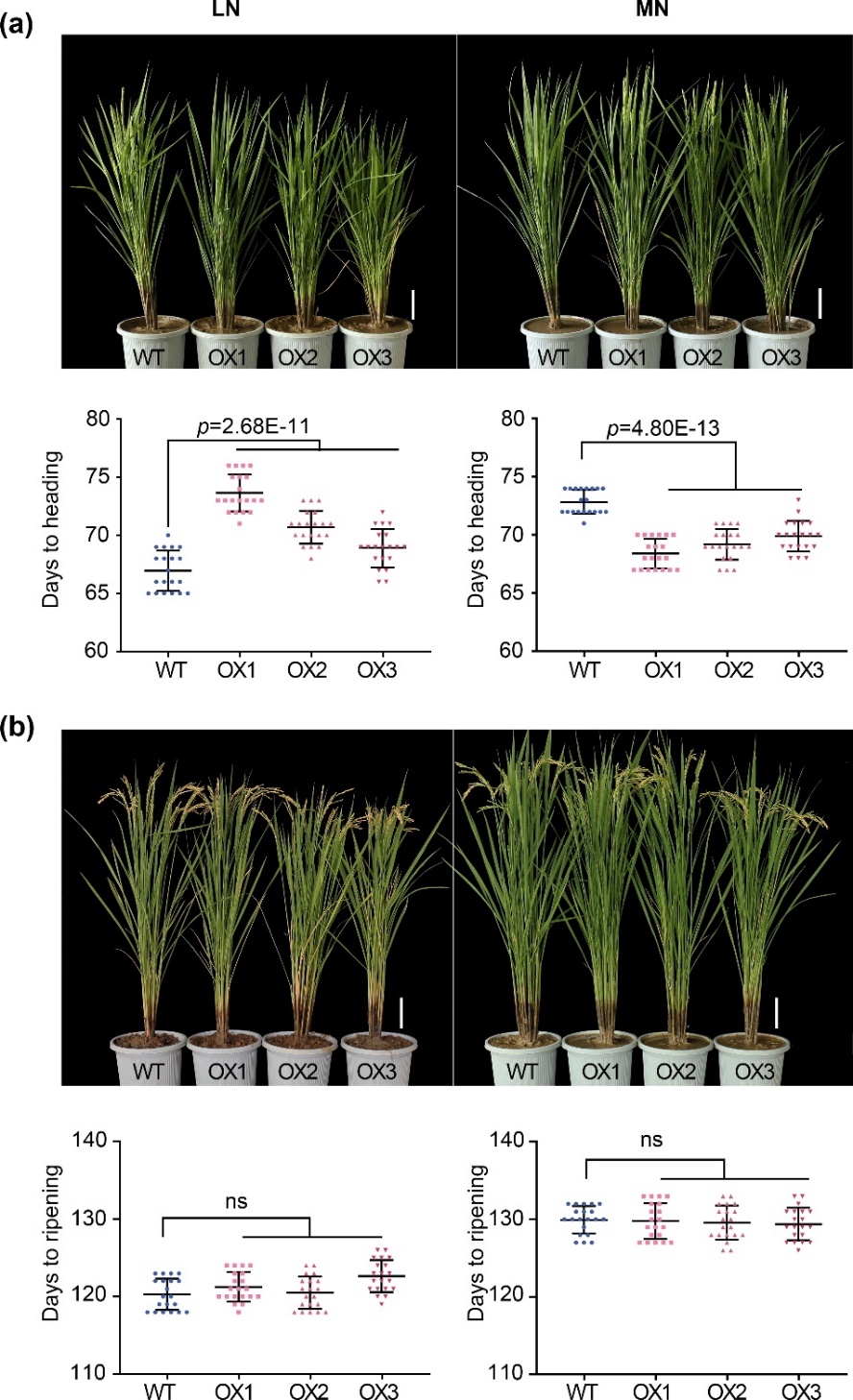


**Figure S3. The heading and ripening phenotype of WT and Nhd1-OX lines under two N treatments in the field trial of second growth season.**

(**a**) Days to heading (DTH) after transplanting under different N treatments. The day to first flowering in main panicle is defined to the flowering time. (**b**) Days to ripening after transplanting under different N treatments. Scale bar = 10 cm. Bars are means ± SD, and each point shows a specific value of each plant. One-tailed Student’s t-test was used for the statistical analysis.


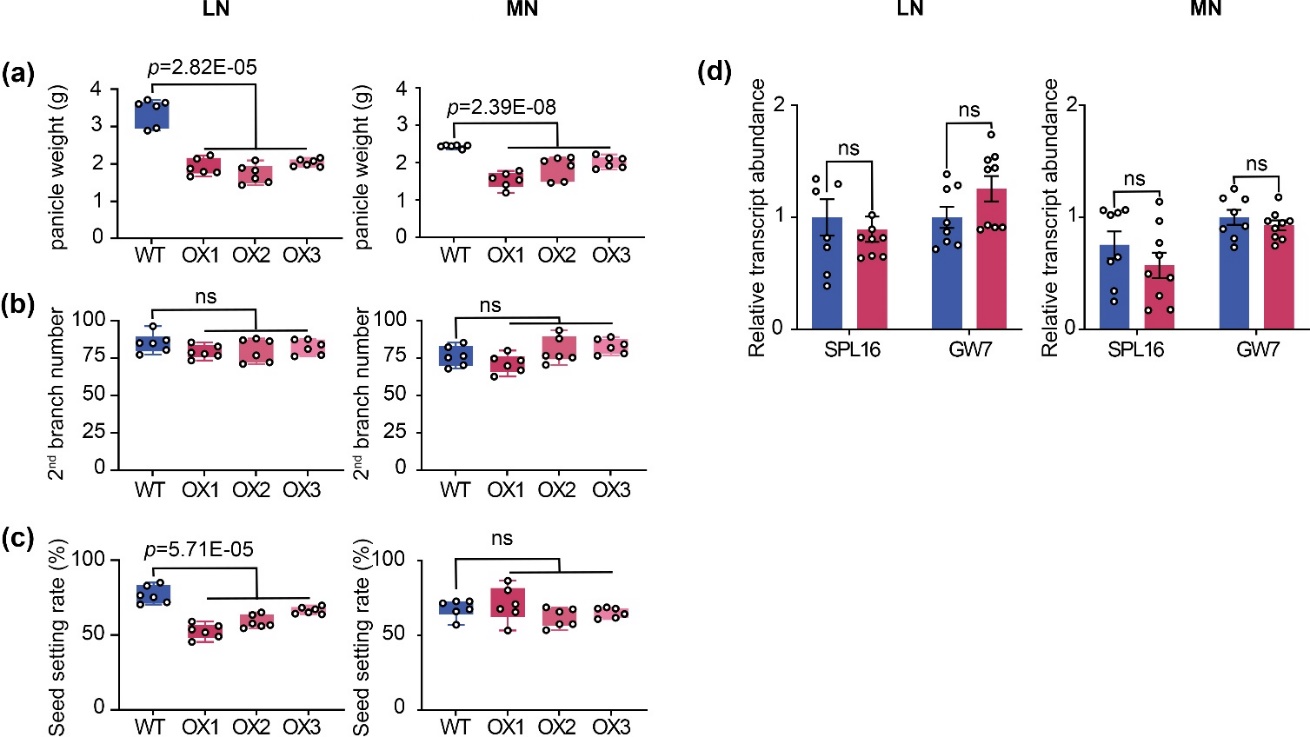


**Figure S4. Panicle characteristics of WT and Nhd1-OX lines under different N supplies.**

(**a-d**) Panicle weight (a) 2^nd^ branch number (b) Seed setting rate (c) relative abundance of genes related to grain length and width (d) of WT and Nhd1-OX plants under two different N treatments. The relative abundance was calculated using the 2^–ΔΔCT^ method. Bars are means ± SD, and each point shows a specific value of each plant (n ≥ 6 plants). One-tailed Student’s t-test was used for the statistical analysis. The *p*-values are provided in the figures.


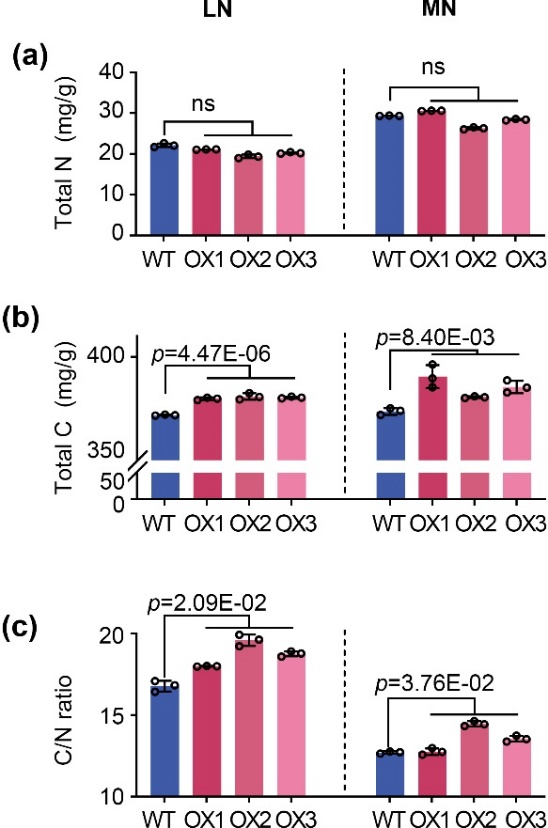


**Figure S5. Total C and N status in the grains of WT and Nhd1-OX lines under two N treatments.**

(**a-c**) Total N concentration (a), total C concentration (b) and ratio of C/N concentration(c) in seeds of WT and Nhd1-OX plants under two different N treatments. Bars are means ± SD, and the black cross represents the average value of each group while each spot shows a mean value of each plant (n = 3 plants). One-tailed Student’s t-test was used for the statistical analysis. The *p*-values are provided in the figures.


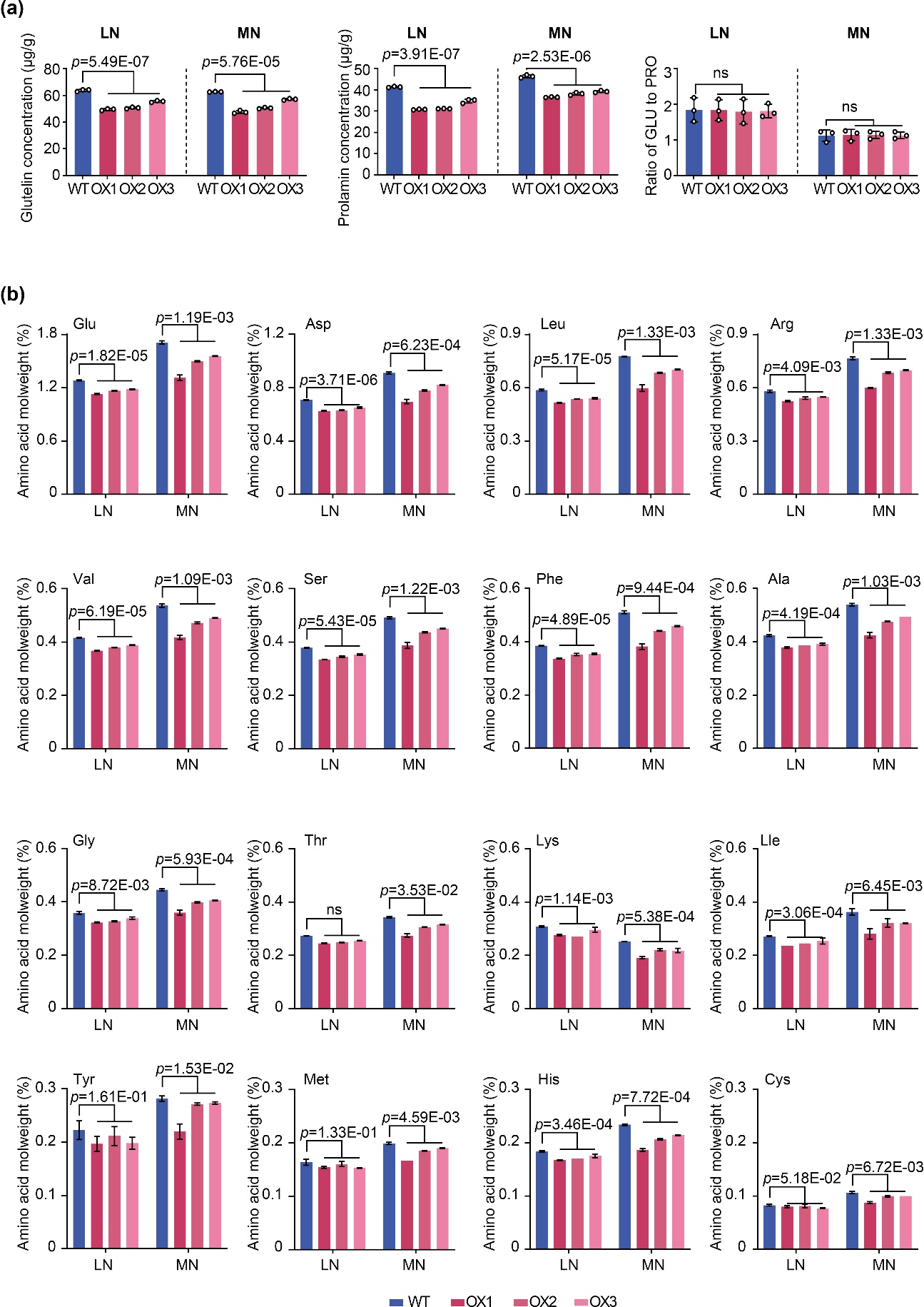


**Figure S6. Quantification of grain storage proteins and main amino acids in WT and Nhd1-OX lines in two different N conditions.**

**(a)** Comparison of glutelin and prolamin concentration, and the ratio of glutelin and prolamin in WT and Nhd1-OX grains in two N conditions. **(b)** Comparison of main amino acids content in WT and Nhd1-OX rice in two N conditions. Bars are means ± SD. One-tailed Student’s *t*-test was used for the statistical analysis. The *p*-values are provided in the figures.


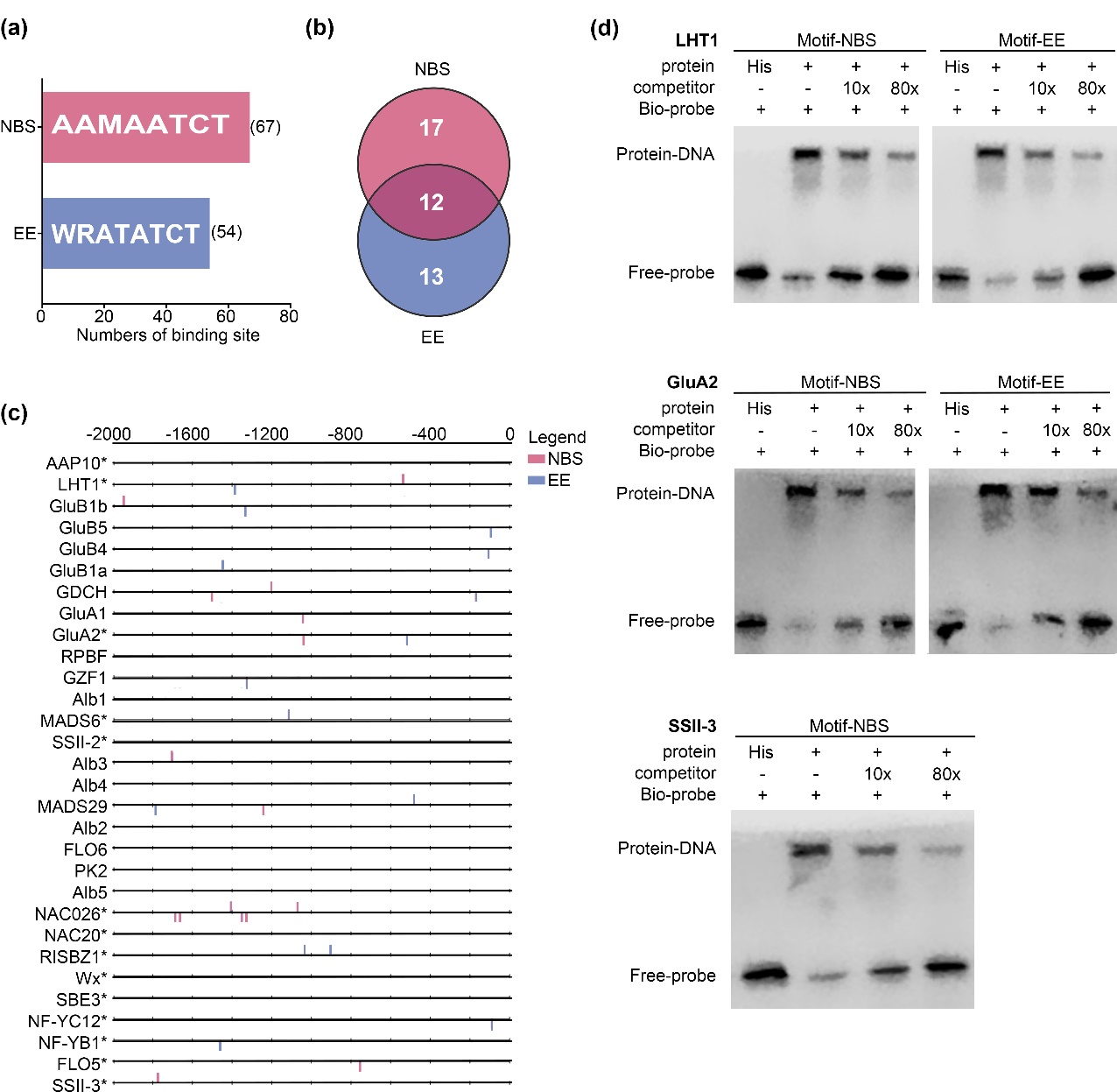


**Figure S7. Nhd1-binding element analysis on the promoter of Nhd1-regulated genes.**

(**a**) The number of NBS and EE cis-acting elements existed in the 2000 bp promoter region of 188 differentially expressed genes (DEGs) between Nhd1-OX and WT. (**b**) The number of genes that contains NBS and EE elements in their promoter among 188 DEGs detected between Nhd1-OX and WT. (**c**) Distribution of Nhd1 and EE elements in the promoter of key genes involved in carbohydrate and protein metabolisms. (**d**) EMSA assays to detect the interaction between Nhd1 and its binding motifs in the promoter of *OsLHT1*, *OsSSII3* and *OsGluA2.*


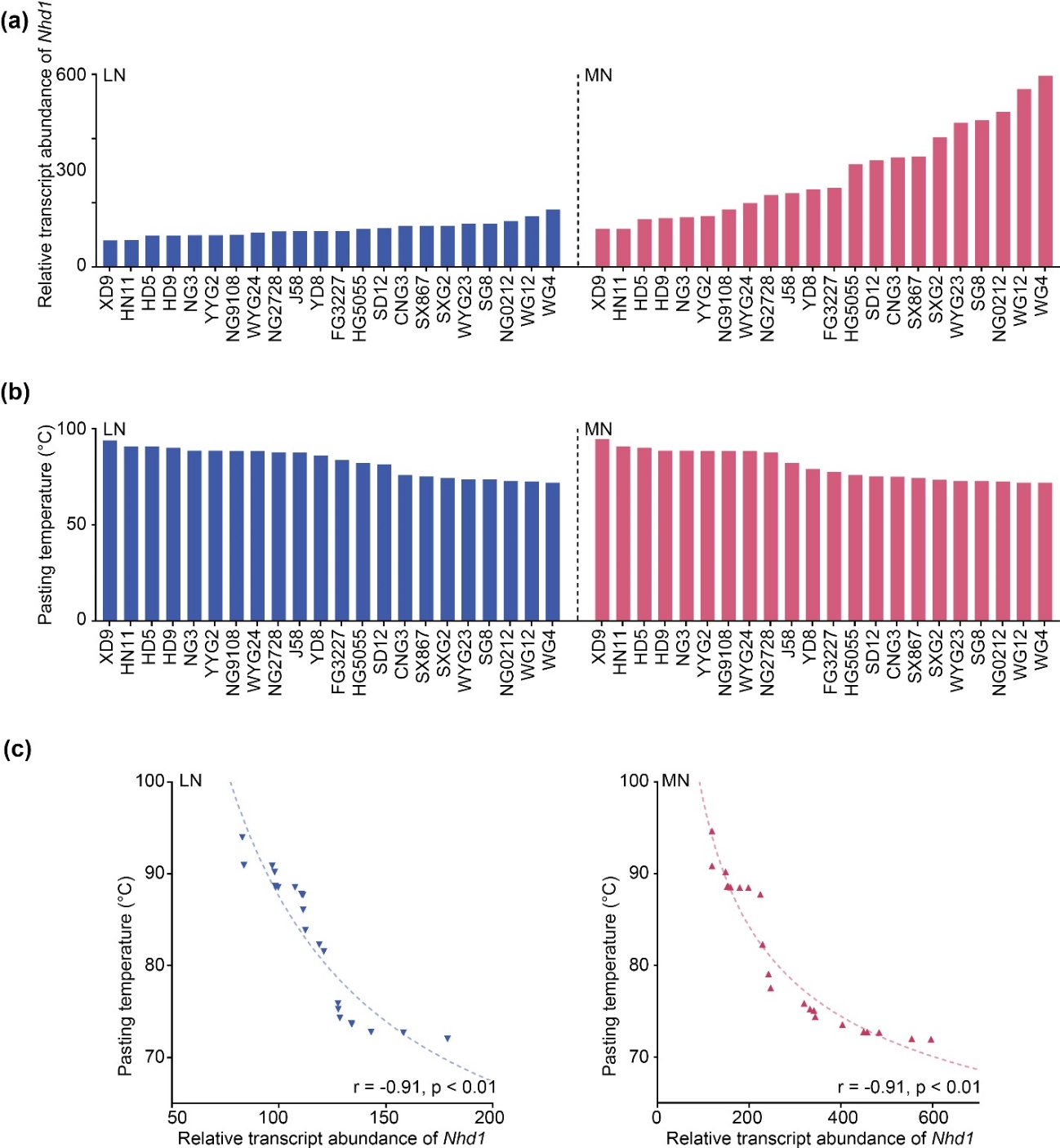


**Figure S8. Correlation between *Nhd1* transcript abundance and pasting characteristics in local main rice cultivars.**

(**a, b**) The *Nhd1* relative transcript abundance (a) and pasting temperature (b) of 22 main rice cultivars in Jiangsu Province under different N conditions. (**c**) The correlations between *Nhd1* expression and pasting temperature under two different N conditions based-on the data in (a) and (b). The line represents the correlation fitting curve, and *p*- and r- value of the curve have been given in the figure.


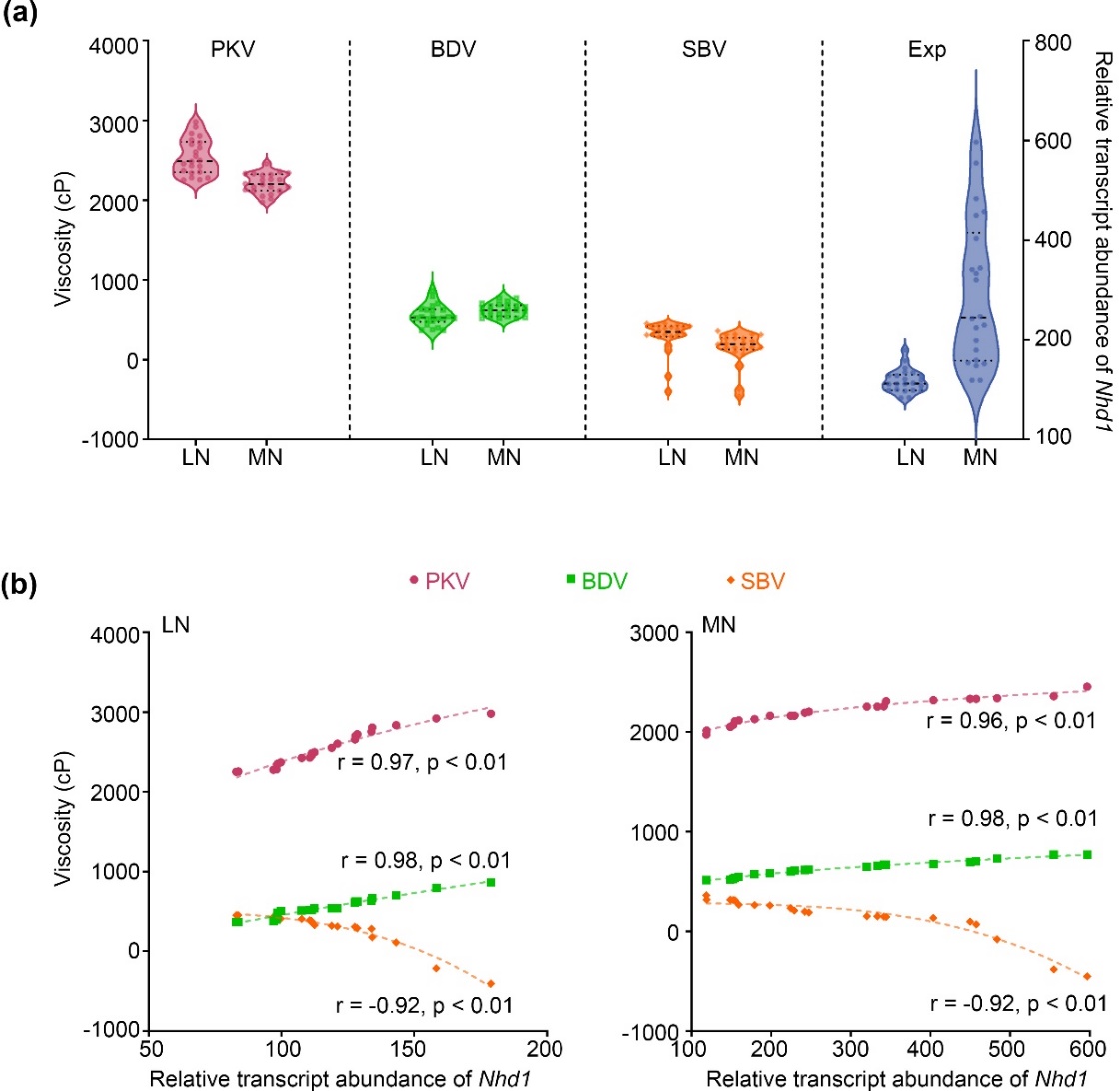


**Figure S9. Improving rice pasting characters by enhancing native promoter-controlled expression of *Nhd1*.**

(**a**) Pasting characters (including PKV, BDV, SBV) and *Nhd1* expression of 22 main rice cultivars in Jiangsu Province under two different N treatments. (**b**) The correlations between *Nhd1* expression and pasting characters based on the data in (a) under two different N treatments. PKV, peak viscosity; HPV, hot paste viscosity; CPV, cool paste viscosity; SBV, setback viscosity (SBV=CPV−PKV); BDV, breakdown viscosity (BDV=PKV−HPV); The line represents the correlation fitting curve, and *p*- and r- value of the curve have been given in the figures.
